# Convergent latitudinal erosion of circadian systems in a rapidly diversifying order of fishes

**DOI:** 10.1371/journal.pgen.1012287

**Published:** 2026-08-31

**Authors:** Daniel B. Wright, Yangfan Zhang, Jacob M. Daane

**Affiliations:** 1 Department of Biology and Biochemistry, University of Houston, Houston, Texas, United States of America; 2 Department of Organismic and Evolutionary Biology, Harvard University, Cambridge, Massachusetts, United States of America; The University of Iowa, UNITED STATES OF AMERICA

## Abstract

Biological clocks allow organisms to anticipate cyclical environmental changes, yet in high-latitude or deep-sea habitats, the diel cues that entrain these rhythms are often seasonally diminished or absent. Fishes of the order Perciformes have rapidly diversified across these arrhythmic ecosystems, raising the question of whether changes to circadian rhythms and biological clock genetic architecture are a component of their evolutionary success. Here, we used a comparative genomic approach to investigate patterns of core biological clock gene loss across 96 perciform and five outgroup species. We found widespread and lineage-specific loss in core clock genes, particularly in the convergently evolving polar and deep-sea suborders Notothenioidei and Cottoidei. This trend of clock gene loss was significantly amplified with higher-latitude species. To determine if these genomic signatures reflect a functional loss of rhythmicity, we performed metabolic phenotyping on three notothenioid species. We found a consistent lack of circadian metabolic oscillations during the late austral fall across all notothenioids, including the sub-Antarctic sister lineage to the cryonotothenioid adaptive radiation, *Eleginops maclovinus*. Experimental data across Perciformes, combined with suborder-wide patterns of gene loss, suggest that a release from circadian constraints occurred early in their diversification, potentially facilitating the repeated expansion of these fishes into polar and deep-sea habitats.

## Author summary

Most animals rely on internal biological clocks to align their behavior and metabolism with predictable environmental cycles. Disruption of these clocks is linked to disease across many species. However, in extreme environments such as the deep sea or polar oceans, the common environmental cues that biological clocks entrain on can be irregular or absent for extended periods. Perciform fishes have repeatedly colonized and diversified in these challenging habitats. What has happened to their biological clocks? By analyzing genome assemblies from 96 perciform species, we identified a widespread and repeated loss of core circadian “clock” genes within this clade. This genetic decay was most pronounced in high-latitude polar species. To assess whether these genomic changes correspond to altered circadian biology, we measured metabolic rates in Antarctic and sub-Antarctic fishes. Both groups lacked detectable metabolic rhythms, including species with no apparent loss of clock genes. Together with data from other perciform species, these findings suggest that some lineages can flexibly abandon circadian rhythms, and that this may have occurred early within specific perciform clades. This flexibility could help to facilitate their success in environments with highly irregular seasonal cycles, where relaxed selection pressures may further drive the degradation of circadian clock components.

## Introduction

Most habitats on Earth experience regular environmental cycles, driven by celestial rhythms such as Earth’s rotation and orbit, which produce predictable patterns of light and darkness, and by lunar forces that govern tidal movements. In response to these rhythms, species have evolved internal biological clocks that enable them to anticipate the optimal time to invest energy in activities such as foraging in synchrony with the cycle of their ecosystem [1]. These endogenous circadian rhythms have been observed across the tree of life, including bacteria, archaea, plants, and metazoans [2,3] and at every stage of an organism’s life cycle [4,5]. To realize the fitness benefits of circadian rhythms, it is essential that biological clocks be calibrated, or “entrained”, to external cues to keep biological rhythmicity synchronized with environmental patterns. The most common environmental cue, also known as a *zeitgeber* (German for ‘time giver’), is light, though food availability, lunar cycles, temperature, and tidal forces have all been reported to influence circadian periodicity [6–8].

The ubiquity of circadian rhythms across biota highlights their central importance in organismal fitness [9–12]. Many genes are regulated by the biological clock; in mammals, up to 43% of all protein-coding genes exhibit circadian expression patterns [13]. Further, many genetic variants have been identified in and around key clock genes that are thought to facilitate adaptation to different photoperiods along environmental gradients [14–18]. Disruption to circadian rhythms in mammals is directly associated with numerous pathologies that would be predicted to negatively impact fitness, including intestinal colitis [19], dysregulation of insulin homeostasis, cardiovascular and cognitive dysfunction [20], and the impairment of fertility [21]. Thus, it is not surprising that mice with mutations affecting clock periodicity have reduced fitness in controlled outdoor experimental enclosures due to negative effects on survival and reproduction [22].

Despite the fitness implications of circadian rhythms, many species have evolved in environments where common environmental zeitgebers are absent or seasonally disrupted and yet thrive in these habitats [23]. High-latitude polar ecosystems experience extreme seasonal variation in periodic cues such as the light/dark cycle that typically entrain circadian rhythms [24]. These extreme shifts in diel periodicity are considered a barrier that challenges poleward-migrating species that otherwise depend on regular circadian rhythms [25]. Polar vertebrates exhibit a spectrum of adaptations to extreme high-latitude light/dark cycles, including sustained circadian entrainment through polar summers and winters, seasonal arrhythmias, and ultradian or free-running rhythms often driven by non-photic cues [24].

The order Perciformes (*sensu stricto*), which contains about 9% of all teleost fish species (~3,300) [26], is overrepresented in high-latitude environments, comprising ~66% of high-latitude fish diversity and encompassing four of the most rapidly diversifying marine clades: Cryonotothenioidei, Sebastidae, Zoarcidae, and Liparidae [27]. These perciform radiations often involve diversification along a depth axis [28], with deep-sea invasions in Perciformes also correlated with latitude [29]. Depth adaptations in this group are extreme; camera traps and trawl surveys have identified perciforms among the most abundant fishes at hadal (> 6,000 meter) depths [30–33]. These deep invasions are attributed in part to reduced stratification of temperature and pressure at high latitudes, which lowers temperature-related physiological barriers to depth invasions [29]. However, another shared but underappreciated feature of these environments is the seasonal or permanent disruption of common circadian cues, such as the light/dark cycle and prey availability.

Is there a unique genetic or physiological feature that potentiates the successful and rapid diversification of Perciformes in extreme environments? Recent high-quality genome assemblies of notothenioids, hadal snailfishes, and deep-sea eelpouts separately uncovered loss of core clock genes [34–37]. These losses may reflect relaxed selection on biological clock genes in arrhythmic environments, but functional evidence remains limited, and data on circadian rhythms in perciforms are sparse. In field studies of geotagged Antarctic bullhead notothen (*Notothenia coriiceps*) along the Antarctic Peninsula, adults showed no daily movement patterns during polar summer or winter, though activity, heart rate, and metabolism varied by season, suggesting possible circannual rhythms [38]. In three-spined sticklebacks (*Gasterosteus aculeatus*) from Lake Témiscouata, only weak and variable circadian locomotor activity was observed under light/dark cycles, with 82% of individuals arrhythmic in constant darkness [39]. Notably, no diel expression of core clock genes (e.g., *arntl1a*, *clock1b*, *clock2*, *per1b*, *cry1b*) was detected, even under regular light/dark cycles [39]. Similarly, two Arctic freshwater sculpins (*Cottus gobio* and *C. poecilopus*) showed seasonally shifting diurnal and nocturnal activity patterns, with *C. poecilopus* arrhythmic in summer [40], a pattern also seen in the marine shorthorn sculpin (*Myoxocephalus scorpius*) [41]. These findings, collected around distinct measures of circadian rhythms, suggest that flexible or reduced circadian control may be a defining feature of this group.

It remains unclear whether disruptions to clock genes and physiological rhythms are restricted to certain lineages or if they are widespread across Perciformes. Here, we analyzed core biological clock gene status in 96 perciform and five outgroup species and experimentally assessed circadian metabolic rhythms in three notothenioid species, including *Eleginops maclovinus*, the sister lineage to the Antarctic adaptive radiation of notothenioids. Our results demonstrate that: (1) clock gene loss is a convergent feature within and between the rapidly diversifying suborders Notothenioidei and Cottoidei, common in polar and deep-sea habitats; (2) the extent of gene loss correlates with latitude; and (3) the absence of daily metabolic rhythmicity in Notothenioidei, even in non-Antarctic species such as *E. maclovinus*, suggests that circadian plasticity evolved prior to their adaptive radiation in a polar environment.

## Results

### Curation of core biological clock components in Perciformes

In vertebrates, the circadian clock is regulated through a transcription-translation feedback loop (TTFL). At the start of this circuit, the transcription factor proteins Circadian locomotor output cycles kaput (Clock) and Aryl hydrocarbon receptor nuclear translocator-like (Arntl, also known as Bmal) form a heterodimer that binds to E-box promoter elements across the genome. The Clock-Arntl heterodimer facilitates the expression of Cryptochrome (Cry) and Period (Per) proteins, which ultimately bind the Clock-Arntl heterodimer and inactivate the complex. This feedback loop takes approximately 24 hours to complete, providing the central mechanism for synchronizing the cellular activity of most organisms with the Earth’s rotation [42].

**Fig 1:**
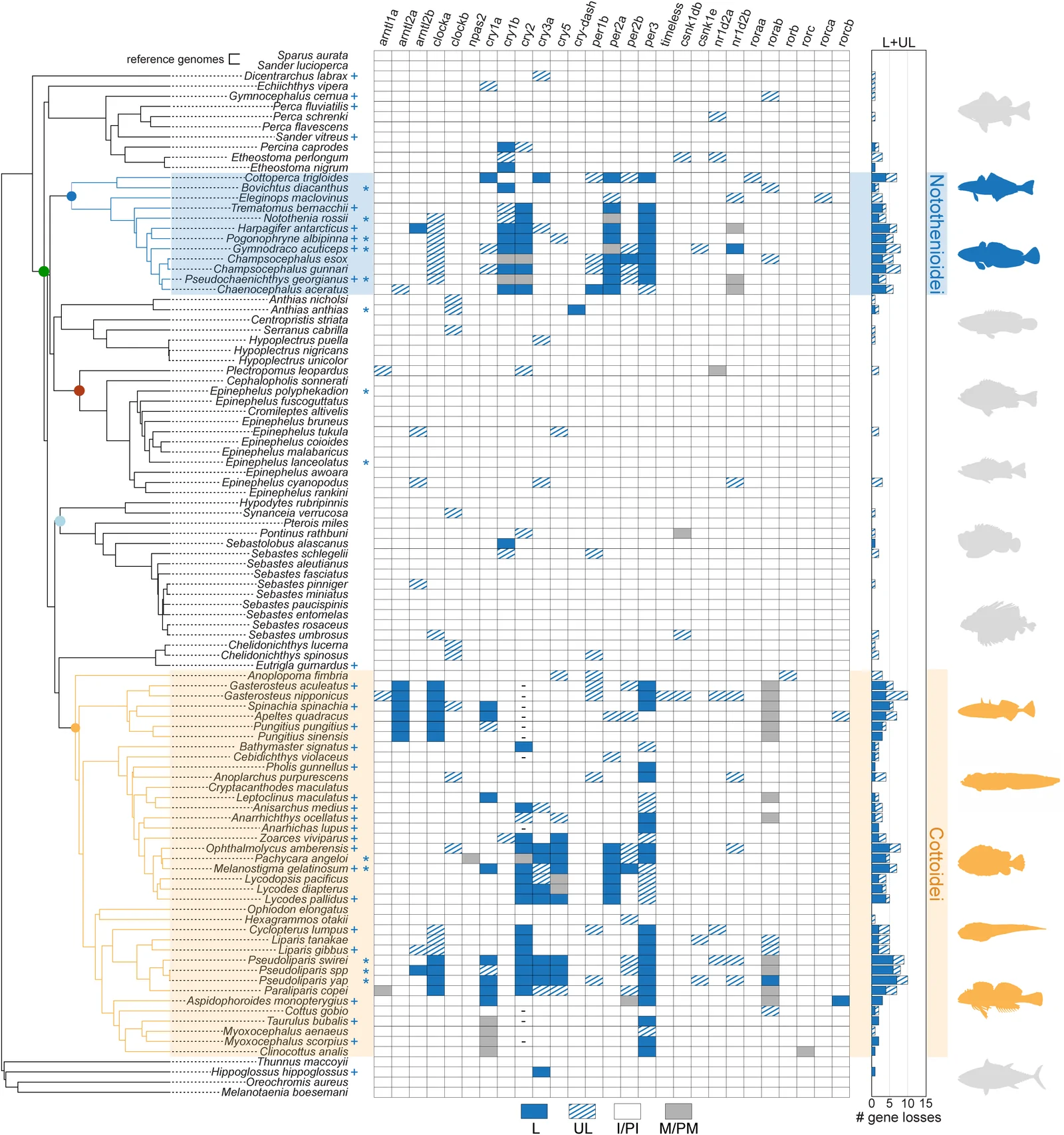
Loss of core biological clock genes in Perciformes. Phylogeny of Perciformes, including five outgroup species, pruned from Rabosky et al [27]. Gene status was inferred from pairwise genome alignments between each species and one of two reference genomes, *Sparus aurata* or *Sander lucioperca*. For each gene in each species, the most complete annotation across both references was used. Losses (L; blue) indicate multiple truncating mutations within the central 80% of the coding sequence (CDS). Uncertain losses (UL; blue hash) reflect a single truncating or deletion variant within the middle 80% of the gene. Gray symbols denote genes that are missing (M) or partially missing (PM), suggesting possible but highly uncertain gene loss or assembly artifacts. White indicates intact or partially intact genes (PI/I; white). White squares with dash (-) indicate instances where Cry2 was identified but out of synteny with the reference genomes. Two clades with elevated gene loss are highlighted: Notothenioidei (blue) and Cottoidei (orange). Ancestral node representing Perciformes marked with a green circle, Serranoidei with a maroon circle, and Scorpaenoidei with a light blue circle. Species whose distribution reaches polar latitudes (≥ 66°) indicated with a + and species whose mean depth ≥ 1000m indicated with a *. Bar plot on the right shows the count of losses (solid blue) and uncertain losses (blue striped) for each species.

**Fig 2:**
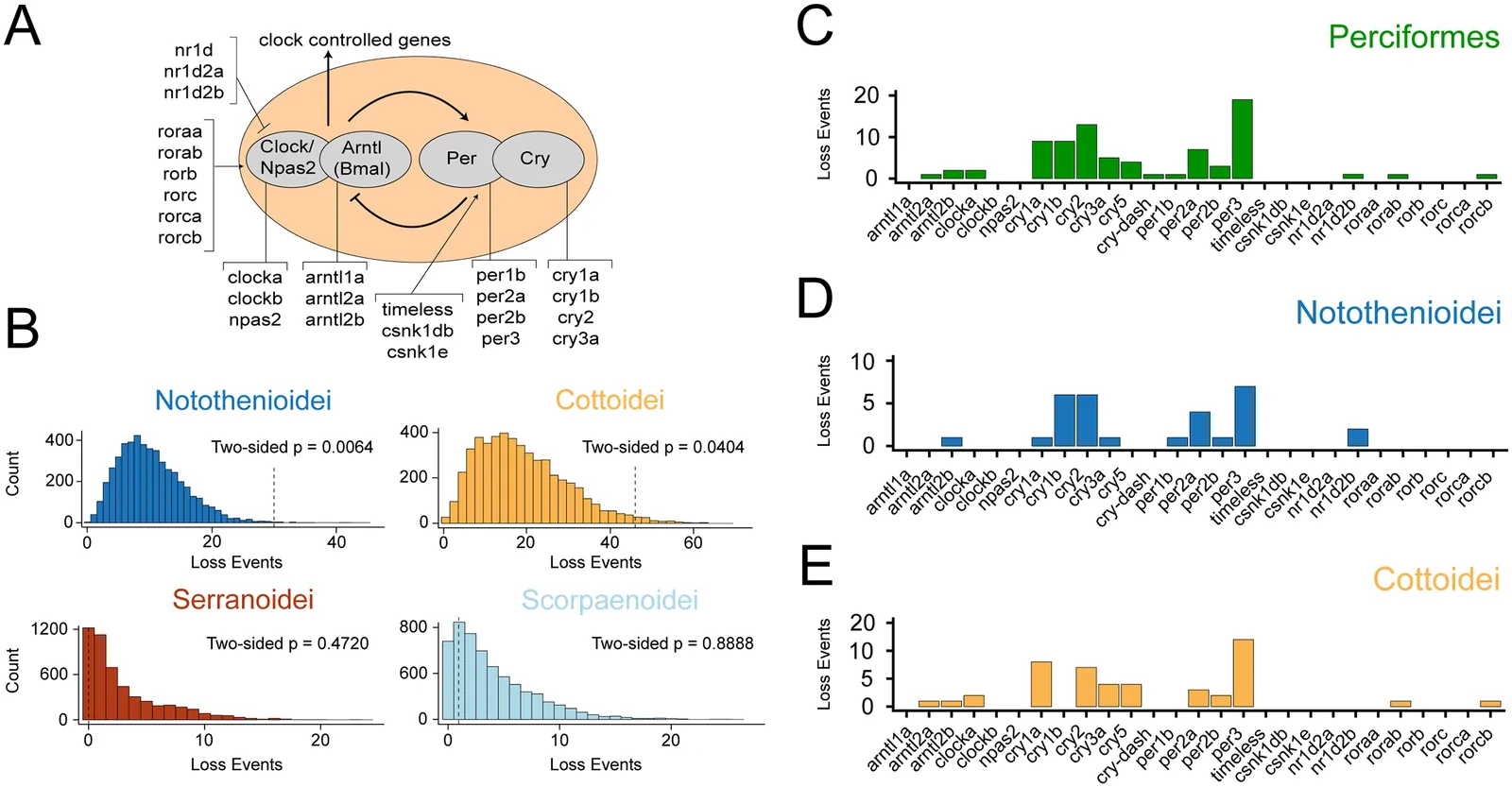
Enrichment of biological clock gene losses in Notothenioidei and Cottoidei. **A)** Schematic of the core biological clock. **B)** Histograms of the distribution of 5,000 randomly sampled subsets of 27 annotated genes for Notothenioidei (top-left), Cottoidei (top-right), Serranoidei (bottom-left), and Scorpaenoidei (bottom right) with two-sided p-value comparing the observed clock loss events (black dashed line) to the null distribution. Bar plots showing the count of clock gene loss events for each clock gene for Perciformes **(C)**, suborder Notothenioidei **(D)**, and suborder Cottoidei **(E)**.

To assess patterns of biological clock evolution in Perciformes, we investigated a panel of 33 well-conserved fish clock-associated genes (Figs 1 and 2A, **S1 Table**) [43]. This set includes paralogs of the core positive transcription factors: the *clock* genes (*clocka*, *clockb*), the *clock* homolog *npas2*, and the *arntl*/*bmal* genes (*arntl1a*, *arntl1b*, *arntl2a*, *arntl2b*) [42,44,45]. We also included the negative regulators of the feedback loop: *period* paralogs (*per1a*, *per1b*, *per2a*, *per2b*, *per3*) and *cryptochrome* paralogs (*cry1a*, *cry1b*, *cry2*, *cry3a*, *cry3b*) [42,46–49]. In addition, we analyzed *cry4*, a photopigment-like cryptochrome implicated in light input and peripheral clock regulation [50], along with *cry5* and *cry-dash*, two cryptochrome family members involved in DNA repair that are light sensitive but are not considered core components of the biological clock [51,52].

We further included regulatory proteins involved in the post-translational modification of the central Clock/Arntl and Cry/Per feedback loop. Among these are the Casein kinases (*csnk1da*, *csnk1db*, *csnk1e*), which phosphorylate Period proteins to regulate their stability, degradation, and nuclear localization [53–55]. We also examined Timeless, a protein essential for clock function in *Drosophila* where it stabilizes Period proteins in the cytoplasm [56]. Although its role in the vertebrate clock is less well defined, Timeless appears to have circadian functions and may link the clock to other cellular processes, such as the DNA damage response or interactions with cryptochromes [56].

Lastly, we included nuclear receptors that help stabilize circadian rhythms through transcriptional regulation. These consist of Nr1d family members (*nr1d1* [Rev-Erbα], *nr1d2a*, *nr1d2b* [Rev-Erbβ paralogs]), which repress the transcription of *arntl* (Bmal) and *clock/npas2* in a rhythmic manner [57], and the retinoic acid-related orphan receptors (Ror), including *roraa*, *rorab* (Rorα paralogs), *rorb* (Rorβ), and *rorc*, *rorca*, *rorcb* (Rorγ paralogs). These Ror proteins act as transcriptional activators of *arntl* and *clock*/*npas2* genes, often competing with Nr1d repressors to maintain circadian balance [58,59].

### Patterns of biological clock gene loss in Perciformes

To assess the status of biological clock genes across Perciformes, we performed a series of pairwise whole-genome alignments and applied the software TOGA (Tool to infer Orthologs from Genome Alignments [60]) to identify orthologs, detect truncating variants, and annotate syntenic gene regions. Genes were considered lost if there are multiple inactivating mutations (e.g., frameshifts, premature termination codons, exon losses, splice site mutations) within the middle 80% of the gene. Genes in loci with fragmented assemblies, lacking syntenic gene regions, with single mutations within the middle 80% of the gene, or with mutations that leave most of the open reading frame intact were given an uncertain status. Aligning genomes to a reference can introduce reference bias due to incomplete gene annotations, assembly artifacts, or species-specific changes to exon boundaries [60]. To control for such biases, we aligned all genomes to two separate reference assemblies. In total, 101 genome assemblies were aligned to reference assemblies of the pikeperch (*Sander lucioperca*, Percidae, Perciformes) and the gilthead seabream (*Sparus aurata*, Sparidae, Spariformes). To capture a broad view of clock genes across the order Perciformes, we included representative genomes from six of the seven recognized suborders. See **S2 Table** for assembly information.

Of the 33 candidate clock genes, 26 genes were annotated in both reference genomes (*S. lucioperca*, *S. aurata*). Of the seven genes not annotated in these references, one gene, *rorab* (*S. lucioperca*: ENSSLUG00000016495; *S. aurata*: ENSSAUG00010006455), was identified as a previously unannotated gene using a *de novo* annotation approach with Exonerate [61]. For the remaining six genes (*arntl1b*, *cry3b*, *cry4*, *per1a*, *csnk1da*, and *nr1d1*), no orthologs were detected with either TOGA or Exonerate in either reference genome; thus, they are presumed to be absent from the genomes of all perciform species included in the study. The absence of these genes is consistent with observed patterns for other members of Acanthomorpha, though previous studies did not consider all genes included here [42].

We detected an unexpectedly widespread pattern of clock gene loss in Perciformes, totaling 125 instances across the order (Fig 1), which translated to 80 gene loss events across the Perciform phylogeny (Fig 2C). These losses were concentrated in two suborders, Notothenioidei and Cottoidei, while other perciform fishes generally retained intact clock genes or had uncertain gene statuses. At the level of gene families, *cry* and *per* paralogs were significantly contracted across the Perciform phylogeny (p = 0.001 and p = 0.049 respectively). The *cry* and *per* gene contractions were limited to the sub-orders Notothenioidei and Cottoidei (**S1****-****S5 Figs**; **S3 Table**). Contractions of *arntl/bmal*, *clock*, and *ror* gene families were insignificant.

**Fig 3:**
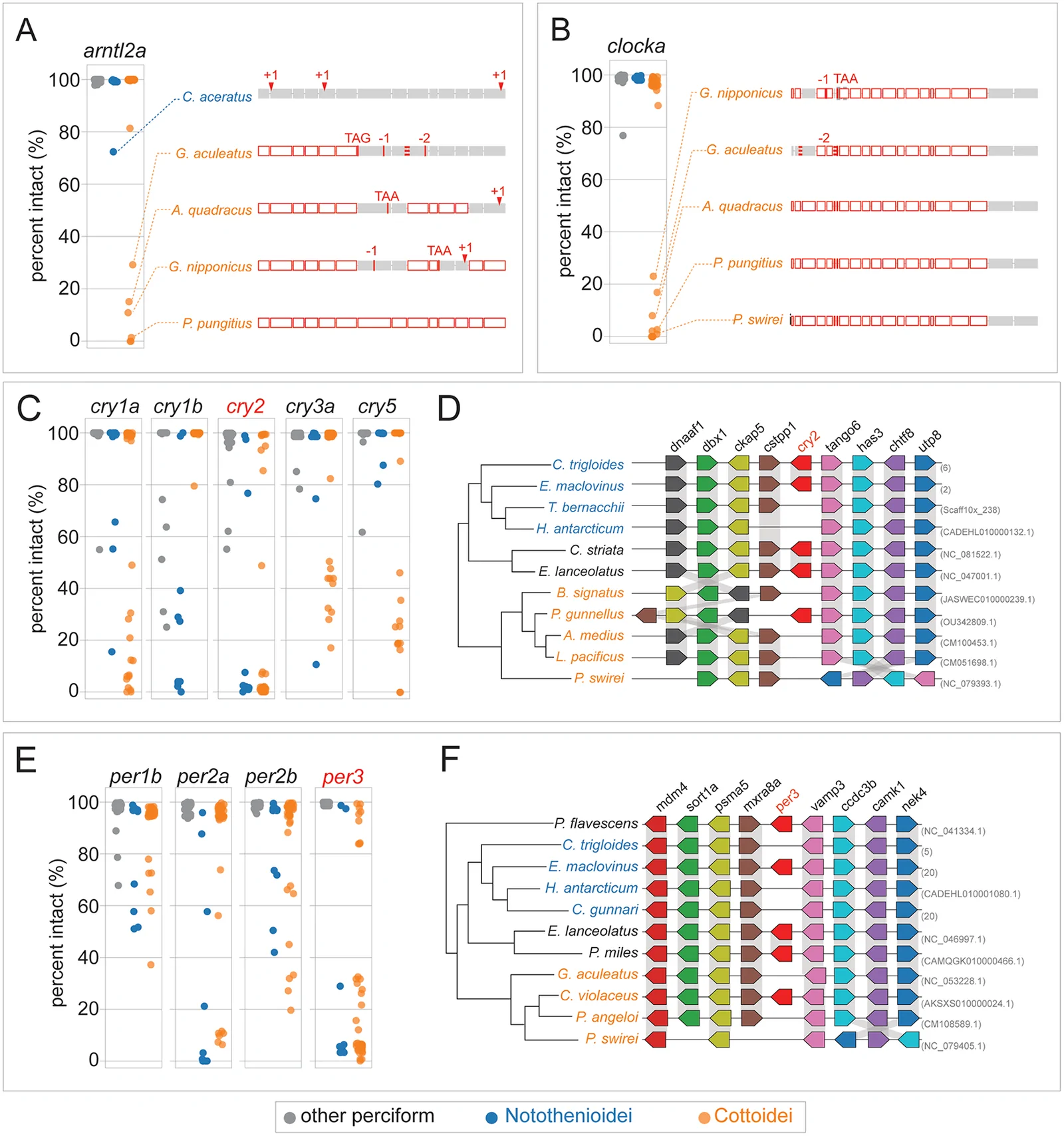
Example mutational variants across central biological clock feedback loop. A–B) Loss patterns of *arntl2* and *clocka*. Plots show the maximum percentage of intact coding sequence (CDS) across all transcript isoforms from pairwise genome alignments to *S. lucioperca* and *S. aurata* reference genomes. Deleted exons are represented with red boxes, present exons with gray boxes. Insertions, deletions, and stop codon are indicated with red text. Species are grouped as Notothenioidei (blue), Cottoidei (orange), or other Perciformes (gray). Representative mutations across each exon for select species are shown to the right. Exons are shaded gray; deleted or missing exons are white with a red outline. Truncating variants are labeled by their type (e.g., frameshifts as +1 or –1), and splice site mutations are marked with red dashed lines at exon boundaries. C–D) Loss patterns of *cryptochrome* genes. Panel D includes a riparian plot showing conservation of the *cry2* syntenic region across Perciformes. E–F) Loss patterns of *period* genes, with a riparian plot of the *per3* locus in panel **F.** See **S6****-****S8 Figs** for further mutation information.

Within Notothenioidei, the number of gene losses ranged from one (*cry1b*) in the thornfish (*Bovichtus diacanthus*) to five in the Antarctic spiny plunderfish (*Harpagifer antarcticus*). The most consistent losses in Notothenioidei occurred in *cry1b*, *cry2*, *per2a*, and *per3*, representing shared losses across the Antarctic clade. Although we initially hypothesized that gene losses would be concentrated within Antarctic lineages due to polar seasonality in light/dark cycles, clock gene loss also occurred outside this group. Notable examples included the channel bull blenny (*Cottoperca trigloides*) with four losses (*cry1a*, *cry3a*, *per2a*, *per3*) and the single loss in *B. diacanthus* described above. Notably, several of these genes lost in sub-Antarctic clades were also independently lost in the cryonotothenioids, including *cry1b*, *per2a*, and *per3*, which show unique mutational patterns across the phylogeny (Figs 3, **S6****-****S7**).

In Cottoidei, we also observed numerous gene losses, ranging from no losses in the basally branching sablefish (*Anoplopoma fimbria*) to seven losses (*clocka*, *cry1a*, *cry2*, *cry3a*, *cry5*, *per3*, *rorab*) in the hadal Yap trench snailfish (*Pseudoliparis yap*) (Fig 1). Within the stickleback family (Gasterosteidae), multiple species share losses of *arntl2a* and *clocka*. Additional lineage-specific losses within Gasterosteidae included *cry1a* in fourspine stickleback (*Apeltes quadracus*) and sea stickleback (*Spinachia spinachia*), and a shared loss of *per3* in the three-spined stickleback (*Gasterosteus aculeatus*), *G. nipponicus*, and *S. spinachia*. Several genes appear to have been lost independently across different Cottoidei lineages, including *cry3a* and *cry5* in both eelpouts and snailfishes; *clocka* in sticklebacks and snailfishes; and *per3* and *cry1a* in sticklebacks and members of the infraorder Cottales.

There were several key distinctions and similarities between loss patterns in Notothenioidei and Cottoidei. In Notothenioidei, gene loss events were concentrated along the Cry/Per axis of the feedback loop (Fig 2D), whereas species in Cottoidei have losses in the Clock/Arntl complex in addition to Cry/Per (Fig 2E). However, several Antarctic notothenioids also showed uncertain gene status for *clocka*. Four genes (*cry2*, *cry3a*, *per2a*, and *per3*) were lost in both Notothenioidei and Cottoidei, representing a convergent loss pattern. All other gene losses appear to be taxon specific. For example, *cry1b* was lost in individual species of Notothenioidei but not in Cottoidei, while *cry5* was lost in some species of Cottoidei but was not lost within Notothenioidei (Fig 2C**-**2E).

While losses were concentrated in Cottoidei and Notothenioidei, a few species outside of these suborders also exhibited gene losses: the common logperch (*Percina caprodes*, gene *cry1b*), Johnny darter (*Etheostoma nigrum*, gene *cry1b*), swallowtail seaperch (*Anthias anthias*, gene *cry-dash*), and shortspine thornyhead (*Sebastolobus alascanus*, gene *cry1b*).

Eleven genes show no clear losses in any perciform species (Fig 2C), including *arntl1a*, *clockb*, *npas2*, *timeless*, *csnk1db*, *csnk1e*, *nr1d2a*, *roraa*, *rorb*, *rorc*, and *rorca*. However, several of these genes have uncertain status in some lineages, which may indicate potential loss of function. Additionally, while many species have lost one of two *cry1* paralogs, there was no conclusive evidence of any individual species losing both *cry1a* and *cry1b*. For example, cryonotothenioids have lost *cry1b* but retain *cry1a.* Conversely, several species have independently lost *cry1a* but not *cry1b*, including *C. trigloides*, the daubed shanny (*Leptoclinus maculatus*), and the limp eelpout (*Melanostigma gelatinosum*).

**Fig 4:**
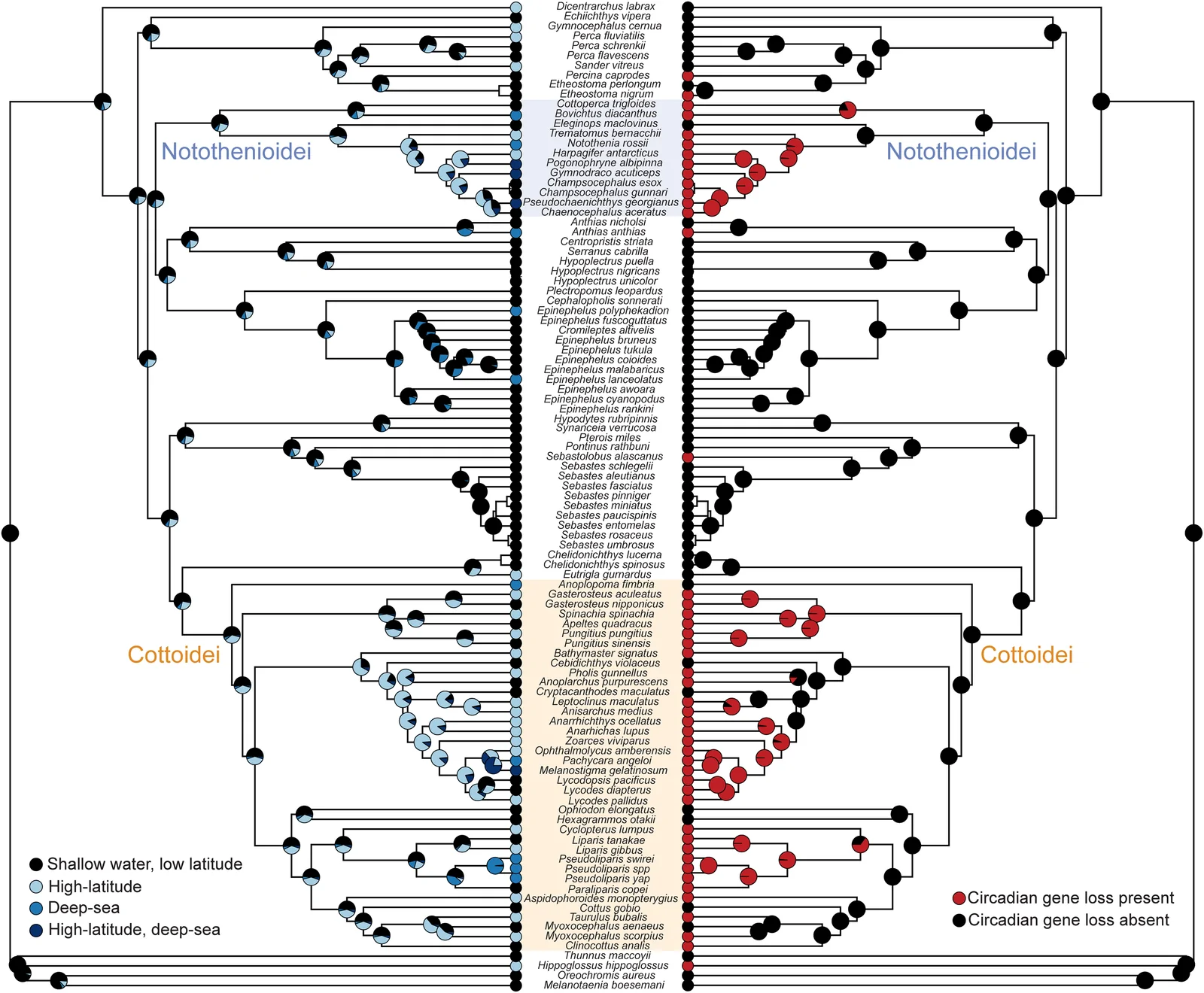
Convergent loss of clock genes across habitat transitions in Perciformes. On the left, a Stochastic character mapping (SCM) of habitat with deep-sea (light blue) defined as a maximum depth of ≥ 1000m, high latitude (dark blue) as a maximum latitude ≥ 66°, or both (purple). On the right, SCM of clock gene loss across the Perciform phylogeny. Loss of at least one biological clock gene is indicated in red, with the absence of gene loss in black. Suborders of Perciformes and the ancestral node connecting Notothenioidei and Cottoidei are labeled.

The losses observed in perciform clock genes occur at a higher rate than would be expected in a random gene sampling. In a distribution of 5,000 randomly sampled subsets of 27 annotated genes, clock gene loss events were significantly more common than expected based on the overall loss patterns for genome assemblies of Notothenioidei, which had 30 clock gene loss events (mean 10.26 ± 5.29, two-sided p < 0.0002), and Cottoidei, which had 46 loss events (mean 18.86 ± 10.71, two-sided p = 0.044). By contrast, Serranoidei had zero loss events (mean 3.05 ± 3.48, two-sided p = 0.472) and Scorpaenoidei had 1 loss event (mean 3.97 ± 3.64, two-sided p = 0.888), both with fewer clock loss events than the expected mean for the null distribution of random genes (Fig 2B). The non-perciform species included in this analysis showed virtually no clock gene losses, despite their greater evolutionary distance from the reference genomes, which could theoretically reduce alignment and annotation accuracy. One notable exception was the Atlantic halibut (*Hippoglossus hippoglossus*), which showed a loss in *cry3a*. Intriguingly, the range of *H. hippoglossus* extends into the Arctic (Figs 1 and 4), and this species inhabits the highest latitudes among the outgroup species.

### Mutational profiles in clock genes

We found a range of mutations among genes classified as lost, including specific truncating variants and complete deletions (Figs 3, **S6****-****S8**). In many cases, the mutation patterns suggested shared ancestral loss within specific suborders or genera. For example, the first six exons of *arntl2a* were deleted in all examined sticklebacks (Fig 3A). Other cases show strikingly similar patterns that likely reflect convergent evolution based on the species tree topology [27], such as the loss of the first 19 exons of *clocka* in multiple stickleback species and in the hadal snailfish *P. swirei*. Most genes, however, displayed patterns consistent with independent gene losses from the accumulation of distinct mutations through drift (**S6****-****S8 Figs**). The most complete gene losses were observed in *cry2* and *per3*, where most species lacked any intact exon sequences (Fig 3C**-**3F). Synteny analysis at the *cry2* (Fig 3D) and *per3* (Fig 3F) loci revealed contiguous genome assembly and gene content conservation, supporting loss of these genes. Detailed evidence of loss for genes in the dataset is provided in **S6****-****S8 Figs**.

### Convergent patterns of clock gene loss and habitat use in Perciformes

Given the observed patterns of gene loss, we hypothesized that biological clock genes were lost independently in Notothenioidei and Cottoidei. To test this hypothesis, we used stochastic character mapping to estimate the probability of clock gene loss in ancestral nodes of the Perciform phylogeny given the observed losses at the tips (Fig 4). Clock loss was assigned if tip species had one or more losses (≥ 1 loss) in the panel of 27 analyzed genes. To provide a comparison to transitions into deep-sea or high-latitude environments, we also mapped the extreme ranges of species habitat onto the phylogeny (Fig 4).

Under an irreversible model of gene loss, clock genes exhibit repeated independent losses both between and within the Notothenioidei and Cottoidei suborders. The common ancestor of these suborders, as well as most internal ancestral nodes, is reconstructed with essentially no probability of clock gene loss, indicating that losses arose repeatedly in descendant lineages. Notably, these loss events closely coincide with transitions into deep-sea and polar environments (Fig 4). In most cases, clock gene losses are associated with species occupying extreme habitats, even when closely related congeners from different environments vary in circadian repertoires (e.g., losses in *Anthias anthias* and *Myoxocephalus scorpius*). The concordance between habitat transitions and clock gene loss among closely related taxa supports repeated, independent colonization of extreme environments accompanied by convergent erosion of clock genes. This pattern suggests that habitat and clock gene loss are broadly associated across the phylogeny.

### Clock gene loss is correlated with latitude

**Fig 5:**
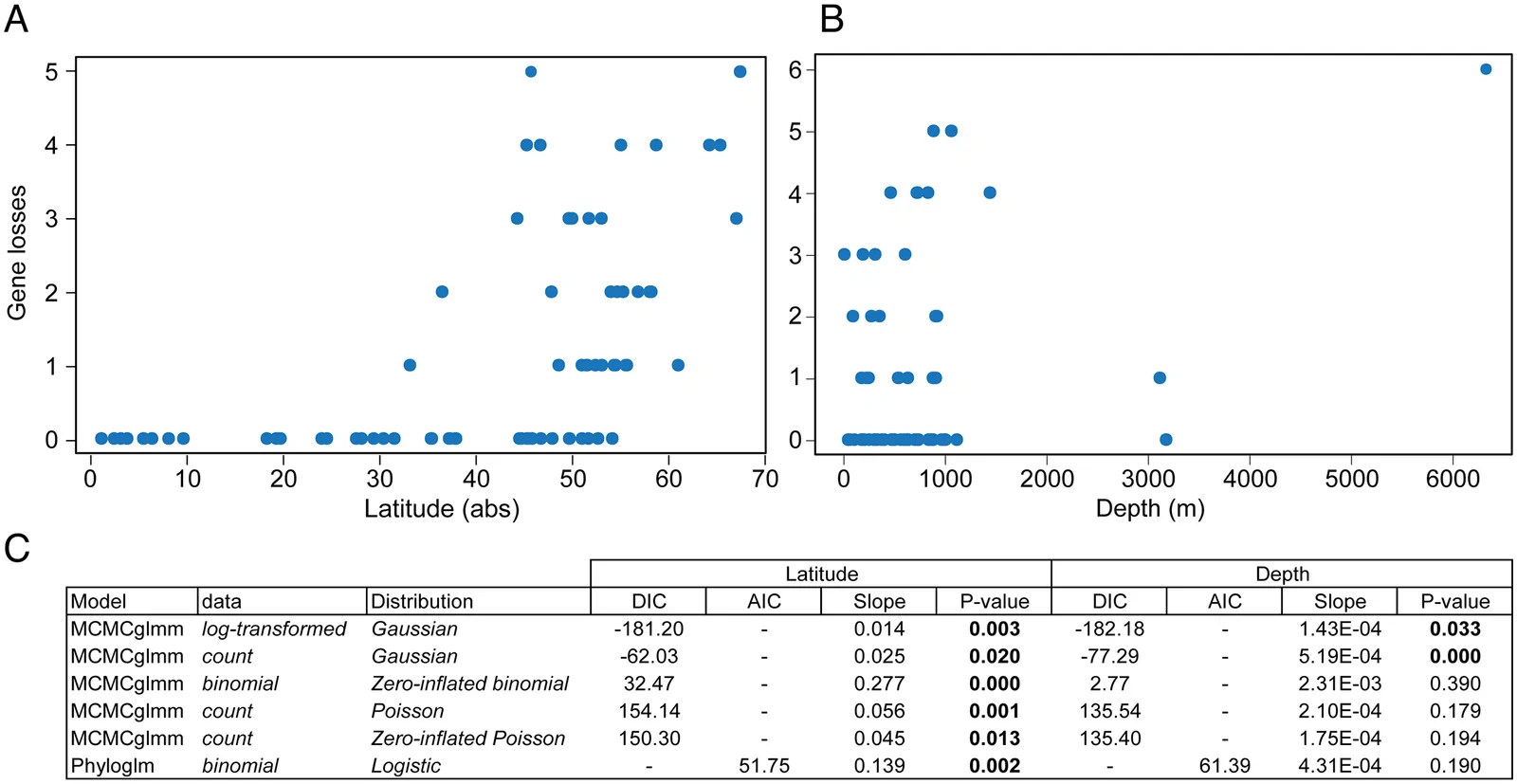
Biological clock gene loss correlates with latitude. Scatter plots show (A) the number of clock gene losses plotted against the absolute value of mean species latitude, and (B) gene losses plotted against mean species depth in meters. Species distribution data are from AquaMaps [98]. Note, deep fishes were removed from the analysis of latitude to avoid potential confounding effects of these two environments. **C)** Results from multiple phylogenetic models testing the association between gene loss and latitude. Statistically significant results are shown in bold.

Given the apparent clustering of gene losses within the Notothenioidei and Cottoidei suborder (Fig 2D,2E), we asked whether there is an association between biological clock gene loss and specific environmental variables. Both Notothenioidei and Cottoidei are abundant in polar and high-latitude regions, as well as across a wide depth gradient [27,29]. We hypothesized that these two environments, high latitude and depth, could be associated with biological clock gene loss due to the seasonally arrhythmic or absent light/dark cycles that characterize these environments respectively (Fig 5A, 5B). Because our dependent variable (count of clock gene losses) is discrete and prone to zero inflation, we tested multiple models and distributions with a combination of counts, log-transformed counts, and binary losses to find the best fit to our data. This process resulted in five mixed models (MCMCglmm) and one phylogenetic generalized linearized model (Phyloglm). All six models investigated show a significant positive relationship between mean latitude and clock gene loss with the Gaussian distribution run on log-transformed losses having the best overall fit (Fig 5C). Two of the models also found a significant relationship between depth and clock gene loss, with the Gaussian distribution applied to log-transformed losses again having the best overall fit.

### Apparent absence of circadian rhythms in the metabolic rate of Notothenioidei

**Fig 6:**
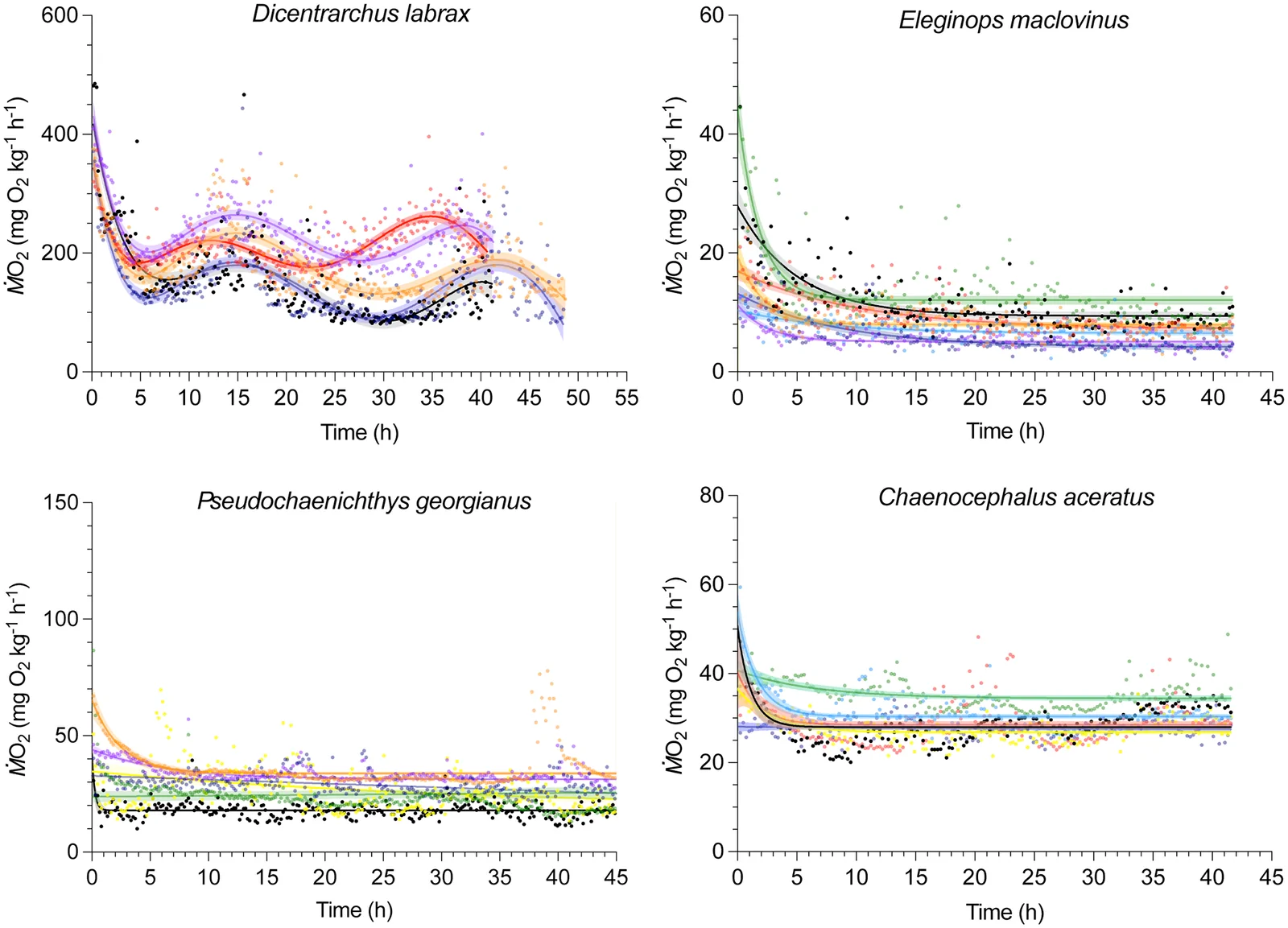
Absence of circadian metabolic profiles in Notothenioidei. Metabolic profiles across two diurnal cycles in four representative species of Percomorpha. *D. labrax* **(A)** is a temperate acanthuriform species. *E. maclovinus* **(B)**, *P. georgianus* **(C)**, and *C. aceratus* **(D)** are notothenioid species within the Notothenioidei clade. Colors represent different individuals. Dots indicate measured aerobic metabolic rates. Solid lines show the fitted regression models, with shaded areas representing 95% confidence intervals. A sixth-order polynomial model was used for *D. labrax*, and a one-phase decay model was applied to *E. maclovinus*, *P. georgianus*, and *C. aceratus*.

The observations of circadian rhythms in Perciformes are sparse but have identified seasonal and inter-individual variability in diel activity patterns [39–41]. Given the widespread patterns of biological clock gene loss, we asked whether these fishes had evidence for circadian rhythms in metabolic rate, and whether changes to circadian rhythms were restricted to polar lineages or if they are also observed in temperate species. Leveraging the quiescent traces of standard metabolic rate (i.e., minimum maintenance metabolism), we analyzed continuous patterns of metabolic rate over a 48-hour window from three species of Notothenioidei. Over two diurnal cycles, the European seabass (*Dicentrarchus labrax*), a temperate acanthuriform species, exhibited a metabolic oscillation at an interval of 25 ± 0.83 hours (Fig 6A). Intriguingly, notothenioid fishes, including sub-Antarctic (*E. maclovinus*) and two Antarctic species (*Pseudochaenichthys georgianus* and *C. aceratus*), all exhibited a lack of oscillation in their metabolic profiles over the two diurnal cycles (Fig 6B**-**6D). Instead, the notothenioids maintained a consistently quiescent metabolic profile at a steady baseline level.

## Discussion

We discovered that biological clock gene loss correlates with latitude and evolved convergently in the perciform suborders Notothenioidei and Cottoidei. The absence of metabolic rhythms in notothenioids links these genomic losses to a disruption in circadian systems. Together with previous studies in perciform fishes, our results highlight an inherent circadian flexibility in both suborders. While the mechanism for gene loss is likely neutral drift under relaxed selection in high-latitude and deep-sea environments, the widespread nature of this erosion of genes and phenotypes across each suborder suggests an ancestral release from strict circadian constraints. Reduced reliance on circadian rhythms may function as a historical contingency for each suborder that is potentially a key component of their success in high-latitude and deep-sea environments.

### Streamlining the perciform biological clock gene set

The gene loss patterns we identified in Notothenioidei and Cottoidei align with previous reports of biological clock gene disruptions in notothenioids [34,35,37], deep-sea snailfishes and eelpouts [36,62], as well as the loss of *per3*, *arntl2a*, and *clocka* in the three-spined stickleback [42,48]. We expand these findings by showing a broader, latitude-correlated pattern of clock gene loss across Perciformes (Figs 1, 2 and 5).

Our data indicate distinct preservation patterns in core biological clock gene paralogs within Perciformes, potentially highlighting reliance on specific clock genes over others (Fig 2C**-**2E). Many infrequently lost genes have pleiotropic functions outside the clock. For example, Cry5, Cry-dash, and Timeless contribute to DNA repair, and retinoid-related orphan receptors (Ror) are involved in immune, metabolic, and developmental processes [52,56,63]. These broader functions likely explain their retention in Perciformes. The consistent presence of *clockb*, *npas2*, and *arntl1a* also suggests essential roles. In contrast, the species-specific retention of either *cry1a* or *cry1b* suggests some functional redundancy between these paralogs. Retention of at least one paralog could be related to residual circadian clock functions or to other essential roles of Cry1 proteins, such as DNA repair [64]. One of the most consistent losses is *per3*, which is absent in multiple species of Notothenioidei and Cottoidei. This gene is frequently lost across teleosts [42], including in Atlantic cod (*Gadus morhua*), northern pike (*Esox lucius*), three-spined stickleback [48], and in many salmonids [49,65], suggesting a diminished role in fish circadian regulation. Still, *per3* remains under intensified purifying selection in Atlantic salmon (*S. salar*), and brown trout (*Salmo trutta*) [49,65]. In contrast, *per1b* and at least one copy of *per2* are relatively well conserved across teleosts [49], including in our dataset (Figs 1, 2C).

The gene *npas2* is retained across all species in our dataset (Figs 1, 2C**-**2E). The Npas2 protein has two PAS (Period–Arnt–Single-minded) domains that bind heme, are sensitive to carbon monoxide, and dimerizes with Arntl (Bmal) in a redox-dependent manner [66,67]. These features may be important for other functions in polar fishes; in notothenioid genomes, there are expansions of oxidative stress-related gene families, which are thought to facilitate adaptation to high cellular oxidative stress in the oxygen-rich Southern Ocean [68].

The patterns of gene loss/conservation support the independent, convergent evolution of clock gene loss between the suborders Notothenioidei and Cottoidei. Both suborders had many losses in the *cry* and *per* complexes, but 5 of the 12 genes lost in Cottoidei were conserved in Notothenioidei (*arntl2a*, *clocka*, *cry5*, *rorab*, *rorcb*) (Fig 2C**-**2E). This finding is further supported by trait mapping, which assigns a low probability of clock gene loss having occurred in the common ancestor of Notothenioidei and Cottoidei (Fig 4). Taken together, our data support the independent, convergent evolution of circadian plasticity at least twice in Perciformes in the suborders with the highest propensity to expand into polar and deep-sea habitats.

This study focused on gene loss within the core biological clock. However, peripherally important clock genes and downstream targets are likely under varied selection regimes. Given the repeated invasion of species of Notothenioidei and Cottoidei into arrhythmic habitats, this is a promising clade for “forward genomic” and genotype-to-phenotype mapping strategies to identify novel circadian-related genetic components through the detection of patterns of convergent molecular evolution (e.g., [69–71]).

### Lack of metabolic rhythms across Notothenioidei and Cottoidei

The three notothenioid fishes measured here show no evidence of metabolic oscillation (Fig 6). In contrast, *D. labrax*, which retains all core clock genes tested (Fig 1), exhibits a robust metabolic rhythm that persists once individuals are placed in constant darkness. Notothenioids, however, maintain a low and steady metabolic rate, consistent with findings of sticklebacks held in the dark and Arctic sculpins in constant lighting [39,40]. This arrhythmicity in laboratory conditions is further consistent with prior observations of the notothenioid *N. coriiceps* held in sea pens [38].

Reports of species entirely lacking circadian rhythms are rare, even in cave environments, and studies often do not exhaustively test all possible zeitgebers or potential rhythmic traits [23]. For example, in the wild, the Mexican blind cavefish (*Astyanax mexicanus*) shows no molecular or behavioral circadian rhythms but can be entrained on light-dark cycles under lab conditions [72]. The Somalian cavefish (*Phreatichthys andruzzii*) has lost the ability to be entrained on artificial light dark cycles [73] despite only one reported clock gene truncation (*per2*, [74]), though remains capable of being entrained on a feeding schedule [75]. There is evidence for rhythms in fish clades found well below the penetration of sunlight (1,000m) (e.g., [76,77]), and many deep-sea invertebrates have circatidal rhythms [78]. Within Cottoidei, an intertidal eelpout (*Zoarces viviparus*) briefly maintains circatidal swimming rhythms in captivity before transitioning to photic cues in the laboratory [79]. Notably, these rhythms exist despite the loss of clock genes within *Z. viviparus* (Fig 1), indicating that many of the gene losses observed within this dataset are not sufficient to fully disrupt biological clocks. However, the retention of circadian entrainment capacity in many of these species may reflect a relatively short evolutionary timescale within arrhythmic habitats that has not yet allowed genetic drift to eliminate entrainment capacity.

Given the persistence of the ability to entrain circadian rhythms in most species, it seems unlikely that notothenioids do not exhibit circadian rhythms of any sort. For example, rhythms may vary seasonally rather than on a daily cycle, or perhaps they were simply not captured by our methods. Additionally, there may be distinctions between benthic and pelagic species. For example, the pelagic Antarctic silverfish (*Pleuragramma antarcticum*) and Antarctic toothfish (*Dissostichus mawsoni*) both show evidence of diel vertical migrations, though the migration of *D. mawsoni* was weaker and more closely followed movements of their prey (*P. antarcticum*) than light intensity [80]. Notably, reports on the extent or existence of diel migrations in pelagic notothenioids seem to vary by method and location [81–83]. Further research is needed to assess the presence and potential seasonality of circadian rhythms across a diverse spectrum of notothenioids, including multimodal analyses of behavior, hormones (e.g., melatonin), gene expression, and testing of non-photic cues (e.g., food, tides) to determine whether these fishes exhibit rhythmicity or are capable of entraining clocks to specific environmental cues.

### Clocks and survival in high latitudes and the deep sea

Fish clades with the fastest speciation rates often include species found in both polar and deep-sea environments, suggesting that there may be underlying shared traits that enable survival in both of these extreme habitats [29]. While most studies focus on cold temperatures or high hydrostatic pressures in these environments, the breakdown of environmental zeitgebers and their impact on engrained circadian mechanisms is also likely an adaptive barrier [25]. Further, in seasonally or permanently arrhythmic environments, responding opportunistically, rather than anticipating cyclic cues, may be advantageous in order to exploit unpredictable conditions. Non-polar and non-deep perciforms show signs of diel flexibility. In addition to the stickleback example mentioned above [39], the slimy sculpin (*Cottus cognatus*; Cottoidei) in Lake Ontario (lat 43°N) shows evidence of diel rhythms in feeding as juveniles at higher water depths (35 m) but has arrhythmic feeding patterns as adults at 75 m [84]. In our dataset, the sub-Antarctic notothenioid *E. maclovinus* had no clock gene losses and only three uncertain losses but shows the same lack of metabolic oscillation as *P. georgianus* and *C. aceratus* (Figs 1, 6). The absence of detectable metabolic rhythmicity in *E. maclovinus*, despite its largely intact gene complement, suggests that phenotypic loss of some circadian functions may have predated the extensive genomic erosion observed in Antarctic lineages. As evidenced by the absence or seasonal plasticity of metabolic rhythms combined with widespread gene losses across multiple perciform species [39–41], this circadian flexibility is an underappreciated component of the invasion of notothenioid and other perciforms into environmental extremes.

### Summary

We find that many core biological clock genes are disrupted in two perciform clades common to high-latitude and deep-sea environments: Notothenioidei and Cottoidei. The extent of gene loss correlates with latitude, with polar species showing a higher extent of loss than temperate lineages. We also show that both Antarctic and sub-Antarctic notothenioids lack daily metabolic rhythms, instead exhibiting steady-state baseline metabolism like that observed in Cottoidei under constant lighting. While high-latitude and deep-sea habitats impose diverse selective pressures, our findings support the idea that altered circadian regulation (through plasticity and genetic erosion) represents a release from an ancestral constraint on strict circadian adherence, potentially fueling their success within arrhythmic habitats. Our study focuses on genes associated with the clock mechanism, but it remains unknown how downstream pathways react to a loss of clock genes, a topic for future research. Because chronic circadian disruption is deleterious in many vertebrates, uncovering how these fishes maintain physiological stability despite reduced circadian control may offer insights into the mechanisms that support resilience in arrhythmic environments.

## Materials & methods

### Ethics statement

All experimental procedures were approved by the University of Alaska Institutional Animal Care and Use Committee (IACUC; protocols 247598–11 and 570217–9). The fish were then held at this temperature for 2.5 weeks. Animal collection, care and use were approved by permit from the Universidad Austral de Chile and UAF IACUC (above).

### Genome alignment, ortholog calling, and determination of gene status

To assess the presence or absence of core clock genes, we employed a whole genome alignment approach that leverages both gene sequence content and syntenic gene context to identify and characterize orthologs across Perciformes. We pairwise aligned a total of 96 perciform and five outgroup genome assemblies to two different reference assemblies, *Sander lucioperca* (GenBank ID: GCA_008315115.1) and *Sparus aurata* (GCA_900880675) (**S2 Table**), using the program *make_lastz_chains* (v 1.0.0) (https://github.com/hillerlab/make_lastz_chains) [85–87].

To identify orthologs, annotate genes, and determine the gene status for the query genomes, we ran TOGA (v 1.1.0-blue) (Tool to infer Orthologs from Genome Alignments [60]) on all pairwise alignment chains. TOGA identifies orthologs between the reference and query genome and analyzes the middle 80% of the query’s open reading frame to determine the gene status. Each gene was classified as intact (I), partially intact (PI), lost (L), or uncertain loss (UL). Briefly, a gene was considered lost if it contained multiple inactivating mutations (e.g., frameshifts, premature stop codons, exon deletions, or splice site disruptions) within the central 80% of the protein-coding sequence (CDS) across all transcript isoforms. Genes with only a single inactivating mutation in this region were categorized as uncertain losses (UL, **S4 Table**), as such cases may result from limited exon conservation, gene annotation errors, or assembly artifacts [88]. Genes with inactivating mutations outside of the middle 80% are considered partially intact (PI). To validate TOGA-derived ortholog assignments using a similarity-based approach, we ran OrthoFinder [89] on TOGA-annotated clock genes for each species in the dataset. We then compared the resulting orthogroups with the orthology predicted by TOGA. This analysis revealed complete concordance between OrthoFinder predictions and TOGA orthology inference (**S5 Table**).

To account for potential alignment, assembly, or annotation artifacts, we cross-referenced genes classified as “lost” by TOGA with the gene expression database Bgee [90] for *Gasterosteus aculeatus*, the only species shared between our dataset and the database. This comparison revealed expression of *cry2*, despite its classification by TOGA as lost in *G. aculeatus*. To identify additional misannotations across our dataset, we re-annotated all clock genes in each genome assembly using Miniprot-0.18 v1.1 [91], followed by reciprocal BLAST+ v2.14.1 [92] searches against a reference proteome. This approach recovered intact *cry2* sequences in 11 species where it was previously labeled as lost by TOGA. We therefore reclassified these genes as intact in our dataset; these corrections are indicated by dashes (–) in Fig 1. These missed *cry2* annotations in TOGA likely result from the gene residing outside the expected syntenic locus in these species compared to both reference assemblies (*S. lucioperca* and *S. aurata*).

Gene annotations based on pairwise genome alignments are prone to reference bias [60]. To address this, we compared the status of each gene in each species relative to both reference assemblies. We used the TOGA orthology inferences generated from a pairwise alignment between the *S. lucioperca* and *S. aurata* genome assemblies to map and compare gene statuses across the different multiple genome alignment datasets. For any discrepancy, we conservatively selected the more intact status between the two reference alignments.

It is also possible that genes are lost within the reference species. To determine the status of clock genes in the reference species, we ran the assembled genomes through the pipeline described above, but with the Nile tilapia (*Oreochromis niloticus,* Cichliformes; GCA_013358895.1) as the reference genome. Notably, aligning to multiple reference assemblies improved the proportion of orthologous genes labeled as intact (I/PI) across the entire dataset, from an average of 67.6% in *S. lucioperca* and 70.9% in *S. aurata* alignments respectively to 73.7% intact (I/PI) in the merged dataset (**S6 Table**).

Genome-wide, we identified orthologs, regardless of intact status, for ≥ 90% of all reference-annotated genes in all species examined, except for the thornfish *Bovichtus diacanthus* (Notothenioidei), which showed slightly lower recovery rates (87% for *S. lucioperca*, 89% for *S. aurata*). Several genes in our candidate list were not explicitly annotated within the *S. aurata* or *S. lucioperca* genome assembly. To determine if these genes were in fact present but unannotated within these species, we used data from a distant relative, the zebrafish (*Danio rerio*, Cypriniformes; GCA_000002035.4). CDS and protein sequence for each candidate gene were extracted from *D. rerio*. We then used the gene model mapper GeMoMa (v 1.9) [93] to infer the gene annotation in our query species and produce an annotation file in GFF format with regions that are a likely match to the reference gene. We then used Exonerate (v 2.2.0) [61] to validate the match between regions annotated by GeMoMa and the protein sequence of the clock genes. Any query genes that were not identified in the analysis were considered missing and excluded from the study.

We used CAFE5 v1.1 [94] to test for significant contraction of biological clock gene families across Perciformes. For input into CAFE5, we generated gene count matrices with OrthoFinder v2.5.5 [89], then aggregated gene count data into four gene families: Arntl (*arntl1a*, *arntl2a*, *arntl2b*), Clock (*clocka*, *clockb*, *npas2*), Cry (*cry1a*, *cry1b*, *cry2*, *cry3a*, *cry5*, *cry-dash*), Per (*per1b*, *per2a*, *per2b*, *per3*), and Ror (*roraa*, *rorab*, *rorb*, *rorc*, *rorca*, *rorcb*).

### Stochastic character mapping

We used stochastic character mapping to test if loss of clock genes evolved convergently or via descent from a common ancestor and to test how clock gene loss is associated with habitat use [95]. We generated two discrete matrices, one with loss of clock genes as the observed trait, the other with habitat type. Species with ≥ 1 loss (gene status L) among the 27 clock genes included in the TOGA analysis were designated as having clock gene loss (trait present). For habitat, species were designated as high-latitude (range extends ≥ 66° latitude), deep-sea (range extends ≥ 1000m depth), both (high-latitude, deep-sea) or neither (low-latitude, shallow). We analyzed these matrices in context of the species phylogeny using the simMap feature in the R package phytools v 2.4.4 [96,97]. We assumed the common ancestor of Perciformes comes from a shallow, low-latitude environment with an intact biological clock gene repertoire and fixed the prior probability (π) at 1 for shallow, low-latitude with no clock gene loss. For the clock gene loss analysis, we used a custom rate model that allowed transition from intact to lost for clock genes but did not allow for their recovery. For habitat, we fit an equal-rate (ER), all rates different model (ARD), and symmetric rates model (SYM) using the make.simmap function with 5000 simulations, then summarized the fit of each with fitMk. The ARD model had the lowest Akaike information criterion (AIC) and was used to estimate posterior probability for ancestral nodes. Data were visualized using the plot function in R.

### Enrichment of clock gene losses in perciform genomes

To determine if Perciformes are enriched for biological clock gene losses, we generated a dataset of 5,000 randomly selected subsets of 27 annotated genes. For each gene, we mapped observed species states onto the tips of the phylogeny and inferred ancestral states using an irreversible-loss assumption. Internal nodes were assigned as intact if any descendent lineage retained the intact state, treating shared losses among related taxa as a single evolutionary event (hereafter referred to as a “loss event”). We ran the analysis for Notothenioidei, Cottoidei, Serranoidei, and Scorpaenoidei (Fig 2B). The comparison between re-sampled and observed data for biological clock genes was assessed using a two-sided empirical p-value.

### Correlation of clock gene loss and the environment

To test if there is a statistical relationship between clock gene loss and latitude or depth, we fit multiple models to our dataset. We used a count of clock genes with a status of “lost” as our dependent variable and latitude or depth as our explanatory variable. We sourced latitude and depth data from the website AquaMaps (aquamaps.org), which aggregates species collection data from multiple biodiversity databases [98]. We calculated a mean latitude and depth from all observations for the 79 perciform species for which data were available (**S7 Table**). We used the absolute value of latitude in our analysis to account for species in both hemispheres. We fit multiple models to our data to account for a discrete dependent variable and the possibility of zero-inflation. We first fit a general linearized model to the data using the R package phyloglm (v 2.6.5) and used a variance-covariance matrix derived from our phylogeny to account for evolutionary history [99–101]. We converted loss counts to binary (zero for no lost genes, one for one or more lost genes) and fit a Poisson distribution. To account for the possibility of zero-inflation and to test additional distributions while still accounting for the evolutionary relationship, we also used a Bayesian phylogenetic generalized linear mixed models in the R package MCMCglmm (v 2.36) [102]. We ran the MCMC for 500,000 iterations with a 10% burn-in and a thinning interval of 40. For all models, we used weakly informative priors (V = 1, nu = 0.002) to minimize their influence on posterior probabilities. To control for the presence of zeros in our data, we tested models with standard counts, log-transformed counts, and binary losses. We used zero-inflated Poisson (ZIP) and standard Poisson distributions with loss counts, a Gaussian distribution with log-transformed losses and a count of losses, and zero-inflated binomial (ZIB) for binary losses (**S9 Fig**, **S8 Table**).

### Collection and housing of experimental animals

Notothenioid species were collected from regions of Low Island (63° 25′ S; 62° 10′ W) and Dallmann Bay (64° 10′ S; 62° 35′ W). The fishing gear consisted of benthic otter trawls deployed from US *ARSV Laurence M. Gould* during the austral fall and winter of 2023. Fish were held in circulating seawater tanks onboard the ship. Fish were then transferred to the aquarium at the Palmer Station (US Antarctic Research Program). At the station, the fish were held in tanks with circulating seawater at 0.1 ± 0.5°C. All tanks were equipped with oxygen diffusers and blocks of frozen seawater were added as needed to maintain the temperature. In the austral fall of 2024, juvenile *Eleginops maclovinus* (Cuvier, 1830) were captured in Reloncaví Fjord (12°C). The fish were held in circulating tanks (8°C) at Los Lagos University for two months before their transportation to Laboratorio Costero de Recursos Acuáticos Calfuco (Universidad Austral de Chile). In the laboratory, the fish were acclimated to 4°C to approximate the thermal physiology conditions of Antarctic notothenioids. The temperature acclimation started with the reduction of the water temperature at a pace of ~1.75°C per day.

A cohort of European sea bass (*Dicentrarchus labrax*) was acclimated to laboratory conditions in a 2000-L indoor tank for 2 months, during which they were fed *ad libitum* twice weekly (Le Gouessant, Lambale, France). A sub-cohort of juvenile European sea bass was distributed between two 500-L tanks. These tanks were supplied with open-flow seawater. The dissolved oxygen level was maintained above 90% air saturation (> 8.2 mg L^-1^). After the metabolic rate measurement trial, all fish spent one hour in a recovery aquarium before returning to the holding tank. Fish holding and experimental procedures followed the guidelines of animal care rules and regulations in France (Apafis 2018040916374437).

### Aerobic metabolic rate

We measured the circadian rhythm of the metabolic rate using an automatic intermittent-flow aquatic respirometry system. The respirometry system measured four individuals simultaneously, each within their own chamber. Hence, the metabolic profiles were obtained for every individual. The automation features of the system enabled undisturbed and continuous measurements for approximately 48 hours. To avoid the effects of digestion, spontaneous activities, and movement, the animals were fasted for 48 hours to reach a post-absorptive state. The metabolic rate of the animals was then measured in a dark and quiescent environment. Thus, only the innate rhythm was manifest in the metabolic profiles. The details of the metabolic rate calculation and the metabolic measuring techniques can be found below and in previously published studies using the same type of respirometry system [103–105]. Before the experiment, animals were held in a ~ 12L:12D cycle in the tank that had a cover to reduce visual disturbances, but the complete blocking of the lights was not guaranteed.

The respirometry system used Loligo-type (LoligoSystems, Denmark, loligosystems.com) respirometer chambers. The size of the chamber was matched to the fish size (water volume: fish ratio ~ 27: 1) to optimize detection sensitivity for the change in water dissolved oxygen (DO) concentration inside of the respirometer due to fish oxygen uptake when the respirometer was in the closed mode. All fish were fasted for at least two days before being placed in the respirometer, where the rate of oxygen uptake (ṀO_2_) was continuously monitored for ~two days, and only the fish that were not visually agitated were included in the final analyses. All four chambers were immersed in a temperature-controlled seawater bath, which was connected via a pump (Eheim 600) to a gas exchange column that delivered aerated water to the respirometers in the open mode. DO was maintained above ~85% saturation throughout the protocol.

Background respiration of each empty respirometer chamber was measured for 20 min before and after each trial and found to be negligible relative to the minimum maintenance metabolic rate of notothenioid species (< 1%, where disinfected seawater was used). For European sea bass, the background respiration in 25 °C seawater exceeded this 1% threshold and the fish’s ṀO_2_ was corrected by subtracting the background ṀO_2_ value. Background respiration was minimized by thoroughly disinfecting the entire apparatus with sodium hypochlorite (Performance bleach, Clorox in 1000 ppm) for 30 min (European sea bass study).

ṀO_2_ was continuously and automatically monitored on-line using computer software (AquaResp v.3, Denmark, Aquaresp.com) that processed water DO measurements in the respirometers (a 1 Hz sampling rate) from an optical oxygen probe associated with each respirometer (Robust Oxygen Probe OXROB10, Pryoscience, pyro-science.com). The optodes were calibrated to 0% saturation (water saturated with sodium sulphite and bubbled with nitrogen gas) and 100% saturation (fully aerated water) at the start of each experiment. ṀO_2_ values were calculated whenever the respirometers were sealed. The measurement cycles (flush period, stabilization period and sealed period) were 65-208-600 for *E. maclovinus*; 80-200-330 for *P. georgianus*; 80-200-600 for *C. aceratus*; and 120-60-420 for *D. labrax*.

The slope of the decrease in DO over time met a minimum requirement for linearity (*i.e.*, R^2^ > 0.9) to calculate ṀO_2_). The quality of PO_2_ traces was checked as described by Chabot et al [106]. Metabolic rate measurements are directly calculated from AquaResp software using the conventional sequential algorithm (Eqn. 1).

### Aerobic metabolic rate calculations

Aerobic metabolic rate was calculated with the following equation:

**Sequential interval** Eqn. 1


$$\begin{array}{c} {\dot{\text{M}}\text{O}}_{\text{2}}=\frac{\left[\frac{{d_{DO}}_{\left[i,(i+a)\right]}}{{d_{t}}_{\left[i,(i+a)\right]}}*\left(V_{r}-V_{f}\right)*S_{O}\right]}{\left(t*M_{f}\right)} \end{array}$$ (1)

where units for ṀO_2_ are mg O_2_ h^-1^ kg^-1^, $\frac{d_{DO}}{d_{t}}$ is the change in O_2_ saturation over time, *V*_*r*_ is the respirometer volume, *V*_*f*_ is the assumed fish volume, *S*_*o*_ is the solubility of O_2_ (calculated by AquaResp v.3 software) in the experimental temperature, salinity and atmospheric pressure, *t* is a time constant of 3600 s (per hour), *M*_*f*_ is fish mass, *a* is *t*he sampling window duration (s), and *i* is 1 DO sample forward from the end of previous sampling window at a set sampling frequency of 1 Hz.

### Modeling of the metabolic profiles

The circadian rhythm of *D. labrax* was modeled using a sixth-order polynomial model (Eqn. 2):


$$\begin{array}{c} y={\text{B}}_{\text{0}}+{\text{B}}_{\text{1}}\cdot x+{\text{B}}_{\text{2}}{\cdot x}^{\text{2}}+{\text{B}}_{\text{3}}{\cdot x}^{\text{3}}+{\text{B}}_{\text{4}}{\cdot x}^{\text{4}}+{\text{B}}_{\text{5}}{\cdot x}^{\text{5}}+{\text{B}}_{\text{6}}{\cdot x}^{\text{6}} \end{array}$$ (2)

where B_0_, B_1_, B_2_, B_3_, B_4_, B_5_, B_6_ are the best fitted coefficients.

The circadian rhythms of *Notothenioidei* were modeled using a one-phase decay model (Eqn. 3):


$$\begin{array}{c} y=(y_{\text{0}}-\text{plateau})\cdot \text{exp}(-\text{k}\cdot x)+\text{plateau} \end{array}$$ (3)

where y_0_ is the intercept, plateau is the y value extrapolated at infinite distance at x-axis, and k is the rate constant.

The regression analyses were conducted in Prism v.10 (GraphPad Software, USA, graphpad.com).

## Supporting information

S1 Fig Phylogeny showing *arntl* gene family for Perciformes. Output phylogeny of CAFE5. Nodes and tips are labeled with the inferred gene family size. Significant changes in family size are indicated with an asterisk (*).
(TIFF)

S2 Fig Phylogeny showing *clock* gene family for Perciformes. Output phylogeny of CAFE5. Nodes and tips are labeled with the inferred gene family size. Significant changes in family size are indicated with an asterisk (*).
(TIFF)

S3 Fig Phylogeny showing *cry* gene family for Perciformes. Output phylogeny of CAFE5. Nodes and tips are labeled with the inferred gene family size. Significant changes in family size are indicated with an asterisk (*).
(TIFF)

S4 Fig Phylogeny showing *per* gene family for Perciformes. Output phylogeny of CAFE5. Nodes and tips are labeled with the inferred gene family size. Significant changes in family size are indicated with an asterisk (*).
(TIFF)

S5 Fig Phylogeny showing *ror* gene family for Perciformes. Output phylogeny of CAFE5. Nodes and tips are labeled with the inferred gene family size. Significant changes in family size are indicated with an asterisk (*).
(TIFF)

S6 Fig Example mutational variants across *period* genes. (A) *per1b*, (B) *per2a*, (C) *per2b*, (D) *per3*. Plots show the maximum percentage of intact coding sequence across all transcript isoforms annotated via pairwise genome alignments to *S. lucioperca* and *S. aurata* reference genomes. Species are grouped as Notothenioidei (blue), Cottoidei (orange), or other Perciformes (gray). Representative mutations across each exon for select species are shown to the right. Exons are shaded gray; deleted or missing exons are white with a red outline. Truncating variants are labeled by their type (e.g., frameshifts as +1 or –1), and splice site mutations are marked with red dashed lines at exon boundaries.
(TIF)

S7 Fig Example mutational variants across *cryptochrome* genes. (A) *cry1a*, (B) *cry1b*, (C) *cry2*, (D) *cry3a*, (E) *cry5*, (F) *cry-dash*. Plots show the maximum percentage of intact coding sequence across all transcript isoforms annotated via pairwise genome alignments to *S. lucioperca* and *S. aurata* reference genomes. Species are grouped as Notothenioidei (blue), Cottoidei (orange), or other Perciformes (gray). Representative mutations across each exon for select species are shown to the right. Exons are shaded gray; deleted or missing exons are white with a red outline. Truncating variants are labeled by their type (e.g., frameshifts as +1 or –1), and splice site mutations are marked with red dashed lines at exon boundaries.
(TIF)

S8 Fig Example mutational variants across assorted biological clock genes. (A) *arntl2b*, (B) *nr1d2b*, (C) *rorab*, (D) *rorc*. Plots show the maximum percentage of intact coding sequence across all transcript isoforms annotated via pairwise genome alignments to *S. lucioperca* and *S. aurata* reference genomes. Species are grouped as Notothenioidei (blue), Cottoidei (orange), or other Perciformes (gray). Representative mutations across each exon for select species are shown to the right. Exons are shaded gray; deleted or missing exons are white with a red outline. Truncating variants are labeled by their type (e.g., frameshifts as +1 or –1), and splice site mutations are marked with red dashed lines at exon boundaries.
(TIF)

S9 Fig MCMCglmm trace plots. Trace plots for each of the MCMCglmm models testing correlation between latitude and circadian gene loss.
(TIFF)

S1 Table Biological clock genes used in the analysis. (XLSM)

S2 Table Genome assemblies used in the analysis. (XLSM)

S3 Table p-values for CAFE5 analysis of gene families. (XLSM)

S4 Table Mutation descriptions for genes with status uncertain loss. (XLSX)

S5 Table Orthology comparison between OrthoFinder and TOGA. (XLSM)

S6 Table Breakdown of global gene status (TOGA) by reference genome. (XLSM)

S7 Table Species location data. Latitude and depth data from AquaMaps.
(XLSM)

S8 Table Autocorrelation tables for MCMCglmm models fit to latitude and depth. (XLSX)
