# Supplementary material for: Convergent latitudinal erosion of circadian systems in a rapidly diversifying order of fishes
Source: PLoS Genet. 2026 Aug 31;22(8):e1012287. doi: 10.1371/journal.pgen.1012287 (PMC13557501; doi:10.1371/journal.pgen.1012287)
Supplement: Supplementary file 1 [file pgen.1012287.s018.pdf]

Wright et al. use comparative genomic approaches to examine biological clock genes in the order Perciformes, which have diversified into arrhythmic ecosystems. They find evidence of loss of clock genes, particularly in the suborders Notothenioidei and Cottioidei and for higher-latitude species. The authors then examine continuous patterns of metabolic rate over 48-hours in 3 species of Notothenioidei, finding that 3 Notothenioidei species (including the sub-Antarctic *Eleginops maclovinus* and two Antarctic species, *Pseudochaenichthys georgianus* and *Chaenocephalus aceratus*) exhibited a lack of oscillation in their metabolic profiles.

Overall, the manuscript examines an interesting question in circadian biology and the approach and methods are generally appropriate. The Notothenioidei and Cottioidei show a compelling pattern of circadian gene loss, and the functional data, while limited, provides some support for differences in clock function. However, I think additional biological context is required and additional analyses would strength the manuscript.

Specific comments:

1) Clock gene redundancy: the manuscript briefly touches on redundancy of clock gene paralogs, but I think this needs to be expanded upon given the limited functional support. In many other systems, clock paralogs are lost while rhythms are maintained, and knockdowns in mice demonstrate that rhythms can be maintained even when individual clock genes are absent due to genetic redundancy. Looking at Figure 1, it seems that there are very few cases where multiple paralogs in the core clock show losses in the same species — for instance, while *cry1b* is lost in several notothenioids, *cry1a* appears retained, and no species appears to have lost both paralogs simultaneously. The authors should more explicitly address whether the observed single-paralog losses are likely to be functionally disruptive given what is known about clock gene redundancy.
2) Further delineation of the “uncertain losses” category would provide important biological context for Figure 1. The current presentation groups several distinct situations (fragmented assemblies, single truncating mutations, etc) under a single "uncertain" designation. Breaking these down to report uncertain cases by more specific designations would help with interpretation.
3) Tests for selection: The authors suggest that losses may reflect relaxed selection on clock genes in arrhythmic environments. Can the authors examine this question by explicitly testing whether there is evidence for relaxed selection on clock genes?
4) The authors suggest that the loss of rhythms potentially facilitated the repeated expansion of these fishes into polar and deep-sea habitats. I am not sure this argument is well supported, as it relies on observations of *E. maclovinus* alone. I would think the loss of clock genes could also reflect drift after movement into environments with reduced rhythmicity, particularly given the association observed with latitude. *E. maclovinus* also seems unusual case for driving this argument, as this species has no circadian gene losses as observed in the other species and shows the same oscillation patterns as species with substantial gene loss. This raises questions as to what is driving the apparent metabolic arrhythmia and whether

this instead reflects the experimental conditions rather than evolved differences in clock function.
