# Supplementary material for: Convergent latitudinal erosion of circadian systems in a rapidly diversifying order of fishes
Source: PLoS Genet. 2026 Aug 31;22(8):e1012287. doi: 10.1371/journal.pgen.1012287 (PMC13557501; doi:10.1371/journal.pgen.1012287)
Supplement: Supplementary file 2 [file pgen.1012287.s019.pdf]

We thank the reviewers for their positive and constructive feedback on the manuscript. Below is a point-by-point response to each comment in blue text with excerpts highlighting the recommended and extensive changes to the manuscript's text and figures. We believe these changes have strengthened the manuscript.

Reviewer 1:

1- Orthology results are central to the work, yet only one orthology method is used. Although it is a good method, and well adapted, I suggest confirming the robustness of results with at least one protein similarity graph method, and reporting orthology results from public databases such as Ensembl Compara, EggNOGG, OrthoDB or OMA. In addition to limiting bias from the choice of method, this would limit bias due to the choice of two specific reference genomes.

> The reviewer is correct that orthology plays a central role in this study. We used Ensembl as the original source to identify circadian genes in our reference species (*Sander lucioperca* and *Sparus aurata*) and thus the data does effectively originate from Ensembl Compara annotation of specific clock genes. To validate TOGA-derived orthology predictions using a similarity-based approach, we ran OrthoFinder on all TOGA-annotated clock genes for each species in the dataset and compared the resulting orthogroups with the orthology assignments predicted by TOGA. The OrthoFinder results 100% matched the TOGA predictions for all intact target circadian genes (see table below). Note, some genes are missing species, reflecting genes that are lost in those genome assemblies.
>
> **Table S4 –** Orthology comparison between OrthoFinder and TOGA

Reference GeneID	Gene Name	Concordant orthology / total sequences
ENSSLUG00000003675	cry1a	95/95
ENSSLUG00000002894	cry1b	94/94
ENSSLUG00000012954	cry3a	98/98
ENSSLUG00000023267	csnk1e	101/101
ENSSLUG00000014906	csnk1db	100/100
ENSSLUG00000015161	roraa	101/101
ENSSLUG00000011202	nr1d2a	101/101
ENSSLUG00000019172	per2b	100/100
ENSSLUG00000010714	per1b	101/101
ENSSLUG00000011054	clockb	101/101
ENSSLUG00000000306	timeless	101/101
ENSSLUG00000023039	arntl2b	101/101
ENSSLUG00000022154	rorb	101/101
ENSSLUG00000007521	rorca	101/101
ENSSLUG00000010091	rorcb	100/100
ENSSLUG00000012433	cry-dash	100/100
ENSSLUG00000023767	npas2	100/100
ENSSLUG00000010667	rorc	100/100
ENSSLUG00000025582	arntl1a	98/98
ENSSLUG00000025458	arntl2a	98/98
ENSSLUG00000015034	cry5	98/98
ENSSLUG00000002539	nr1d2b	97/97
ENSSLUG00000022765	per2a	92/92
ENSSLUG00000011628	clocka	93/93
ENSSLUG00000016495	rorab	87/87
ENSSLUG00000011504	per3	72/72
ENSSLUG00000025356	cry2	59/59

‘And we have added the following to the methods section:

> “To identify orthologs, annotate genes, and determine the gene status for the query genomes, we ran TOGA (v. 1.1.0-blue) (Tool to infer Orthologs from Genome Alignments (60)) on all pairwise alignment chains. TOGA identifies orthologs between the reference and query genome and analyzes the middle 80% of the query’s open reading frame to determine the gene status. Each gene was classified as intact (I), partially intact (PI), lost (L), or uncertain loss (UL). Briefly, a gene was considered lost if it contained multiple inactivating mutations (e.g., frameshifts, premature stop codons, exon deletions, or splice site disruptions) within the central 80% of the protein-coding sequence (CDS) across all transcript isoforms. Genes with only a single inactivating mutation in this region were categorized as uncertain losses (UL, **Table S4**), as such cases may result from limited exon conservation, gene annotation errors, or assembly artifacts (89). Genes with inactivating mutations outside of the middle 80% are considered partially intact (PI). To validate TOGA-derived ortholog assignments using a similarity-based approach, we ran OrthoFinder (90) on TOGA-annotated clock genes for each species in the dataset. We then compared the resulting orthogroups with the orthology predicted by TOGA.

This analysis revealed complete concordance between OrthoFinder predictions and TOGA orthology inference (**Table S5**).”

2- I have several comments on the comparison between species and phylogenetic reasoning. Gene loss in the ancestor of a species clade is one single event, which mostly cannot be reversed. Thus: 2a- Gene losses should not be counted in number of genomes without a gene, but in number of monophyletic clades without a gene, which correspond to the number of events of loss. This notably applies to Fig. 2, and to the paragraph starting line 155.

This is a great point. We have adjusted the counting to quantify independent gene losses by mapping presence/absence data onto the species tree and counting each monophyletic loss-clade as a single evolutionary event. This had minimal impact on statistical significance. Below is an updated methods and results excerpt with the new version of Fig. 2. Note, this includes background branch controls in response to comment 2c.

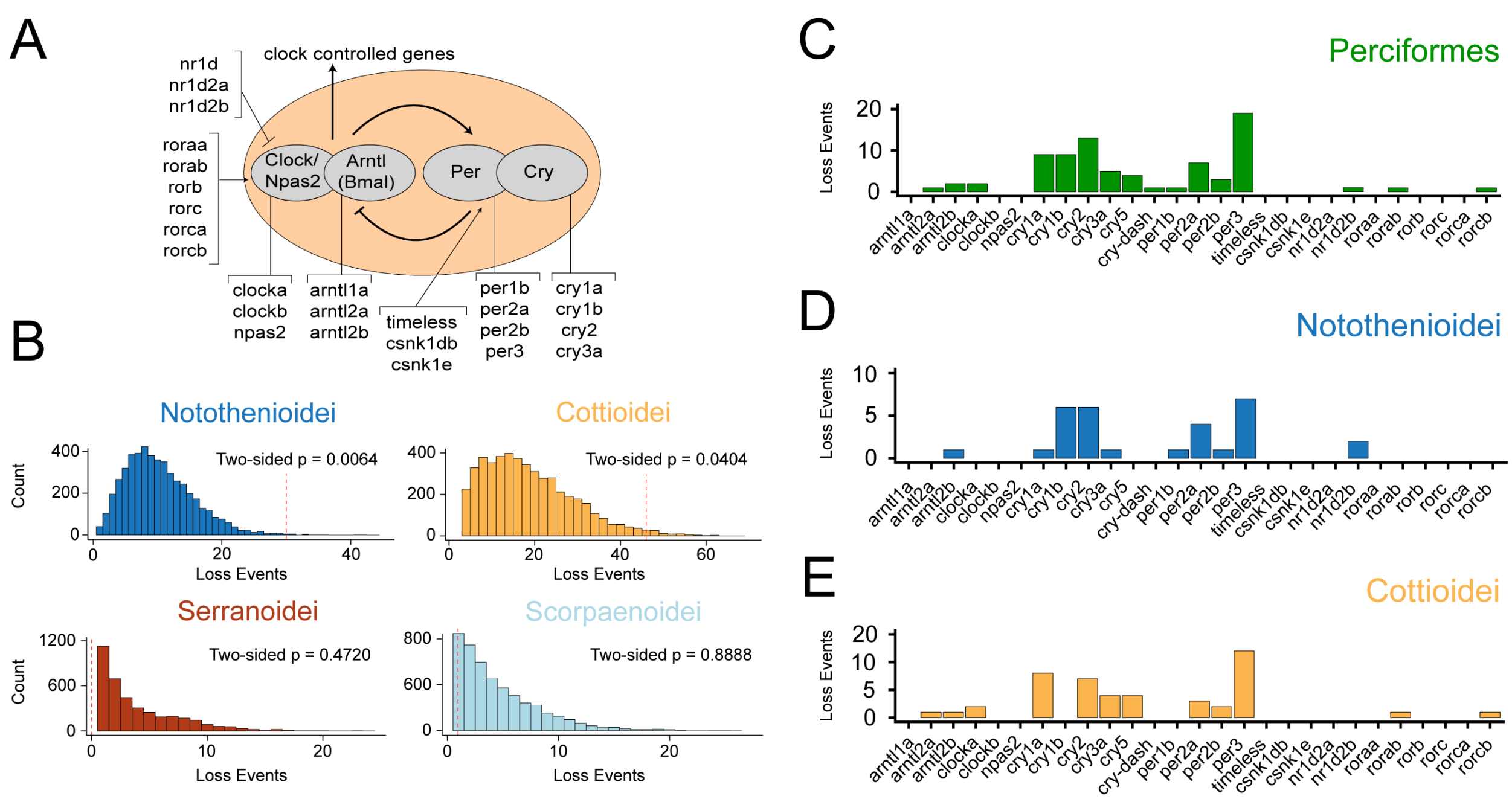

Fig. 2 - Enrichment of biological clock gene losses in Notothenioidei and Cottioidei. A) Schematic of the core biological clock. B) Histograms of the distribution of 5,000 randomly sampled subsets of 27 annotated genes for Notothenioidei (top-left), Cottioidei (top-right), Serranoidei (bottom-left), and Scorpaenoidei (bottom right) with two-sided p-value comparing the observed clock loss events (black dashed line) to the null distribution. Bar plots showing the count of clock gene loss events for each clock gene for Perciformes (C), suborder Notothenioidei (D), and suborder Cottioidei (E).

Methods:

"To determine if Perciformes are enriched for biological clock gene losses, we generated a dataset of 5,000 randomly selected subsets of 27 annotated genes. For each gene, we mapped observed species states onto the tips of the phylogeny and inferred ancestral states using an irreversible-loss assumption. Internal nodes were assigned as intact if any descendent lineage retained the intact state, treating shared losses among related taxa as a single evolutionary event (hereafter referred to as a "loss event"). We ran the analysis for Notothenioidei, Cottioidei, Serranoidei, and Scorpaenoidei (**Fig. 2B**). The comparison between re-sampled and observed data for biological clock genes was assessed using a two-sided empirical p-value."

Results:

"The losses observed in perciform clock genes occur at a higher rate than would be expected in a random gene sampling. In a distribution of 5,000 randomly sampled subsets of 27 annotated genes, clock gene loss events were significantly more common than expected based on the overall loss patterns for genome assemblies of Notothenioidei, which had 30 clock gene loss events (mean 10.26 ± 5.29, two-sided $p < =0.0002$), and Cottioidei, which had 46 loss events (mean 18.86 ± 10.71, two-sided $p=0.044$). By contrast, Serranoidei had zero loss events (mean 3.05 ± 3.48, two-sided $p=0.472$) and Scorpaenoidei had 1 loss event (mean 3.97 ± 3.64, two-sided $p=0.888$), both with fewer clock loss events than the mean for the null distribution of random genes (Fig. 2B). The non-perciform species included in this analysis showed virtually no biological clock gene losses, despite their greater evolutionary distance from the reference genomes, which could theoretically reduce alignment and annotation accuracy. One notable exception was the Atlantic halibut (*Hippoglossus hippoglossus*), which showed a loss in *cry3a*. Intriguingly, the range of *H. hippoglossus* extends into the Arctic (**Fig. 1**), and this species inhabits the highest latitudes among the outgroup species."

2b- For Fig. 4, what biology are the authors representing with a model "which allows for backward and forward transitions"? A more straightforward interpretation of the tree is that each clade with only losses corresponds to one loss event, and every clade with a gene present in even one species implies that the gene was present in the ancestor of this clade. It would be more informative to map on the tree of Fig 4 the number of circadian genes infered lost on each branch.

We had originally selected the ER model as it provided the best overall statistical fit for the data. However, the reviewer is correct that it does not make biological sense that a gene loss would be reverted. We have rerun the stochastic character mapping analysis with a custom loss/gain model that allows for transitions from present to lost, but not from lost to present. **Fig. 4** has been updated along with the figure description and methods section. In

the figure we have added a second phylogeny that maps environmental transitions to deep-sea and high latitude. See response to reviewer 1 comment 10.

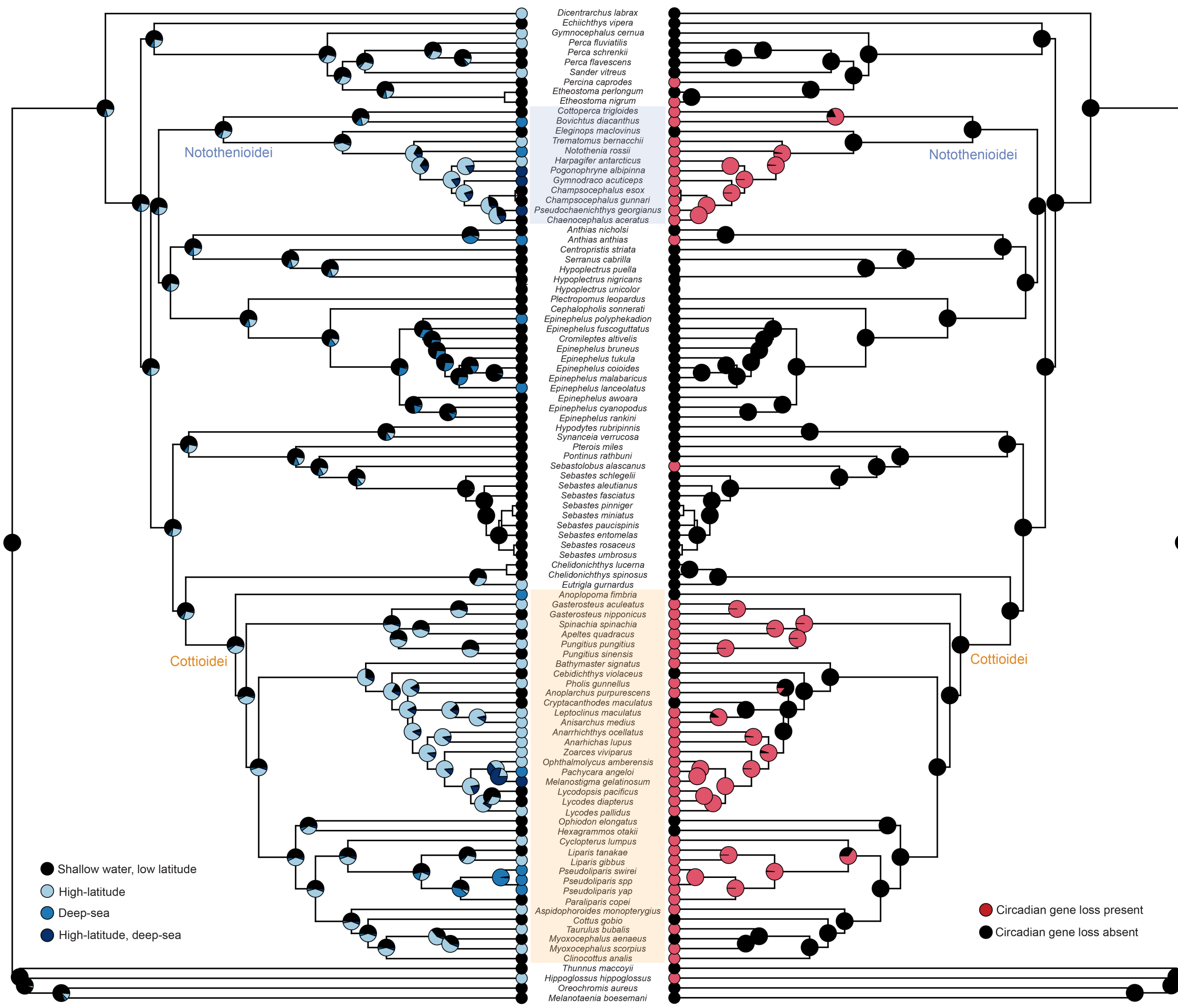

Fig. 4 – Convergent loss of clock genes across habitat transitions in Perciformes. On the left, a Stochastic character mapping (SCM) of habitat with deep-sea (light blue) defined as a maximum depth of ≥ 1000m, high latitude (dark blue) as a maximum latitude ≥ 66°, or both (purple). On the right, SCM of clock gene loss across the Perciform phylogeny. Loss of at least one biological clock gene is indicated in red, with the absence of gene loss in black. Suborders of Perciformes and the ancestral node connecting Notothenioidei and Cottioidei are labeled.

Methods:

"We used stochastic character mapping to test if loss of clock genes evolved convergently or via descent from a common ancestor and to test how clock gene loss is associated with habitat use (96). We generated two discrete matrices, one

with loss of clock genes as the observed trait, the other with habitat type. Species with ≥ 1 loss (gene status L) among the 27 clock genes included in the TOGA analysis were designated as having clock gene loss (trait present). For habitat, species were designated as high-latitude (range extends ≥ 66° latitude), deep-sea (range extends ≥ 1000m depth), both (high-latitude, deep-sea) or neither (low-latitude, shallow). We analyzed these matrices in context of the species phylogeny using the simMap feature in the R package phytools v 2.4.4 (97, 98). We assumed the common ancestor of Perciformes comes from a shallow, low-latitude environment with an intact biological clock gene repertoire and fixed the prior probability (π) at 1 for shallow, low-latitude with no clock gene loss. For the clock gene loss analysis, we used a custom rate model that allowed transition from intact to lost for clock genes but did not allow for their recovery. For habitat, we fit an equal-rate (ER) and, all rates different model (ARD), and symmetric rates model (SYM) using the make.simmap function with 5000 simulations, then summarized the fit of each with fitMk. The ARD model had the lowest Akaike information criterion (AIC) and was used to estimate posterior probability for ancestral nodes. Data were visualized using the plot function in R."

Results:

"Given the observed patterns of gene loss, we hypothesized that biological clock genes were lost independently in Notothenioidei and Cottioidei. To test this hypothesis, we used stochastic character mapping to estimate the probability of clock gene loss in ancestral nodes of the Perciform phylogeny given the observed losses at the tips (**Fig. 4**). Clock loss was assigned if tip species had one or more losses (≥ 1 loss) in the panel of 27 analyzed genes. To provide a comparison to transitions into deep-sea or high-latitude environments, we also mapped the extreme ranges of species habitat onto the phylogeny (**Fig. 4)**.

Under an irreversible model of gene loss, clock genes exhibit repeated independent losses both between and within the Notothenioidei and Cottioidei suborders. The common ancestor of these suborders, as well as most internal ancestral nodes, is reconstructed with essentially no probability of clock gene loss, indicating that losses arose repeatedly in descendant lineages. Notably, these loss events closely coincide with transitions into deep-sea and polar environments (**Fig. 4**). In most cases, clock gene losses are associated with species occupying extreme habitats, even when closely related congeners from different environments vary in circadian repertoires (e.g., losses in *Anthias anthias* and *Myoxocephalus scorpius*). The concordance between habitat transitions and clock gene loss among closely related taxa supports repeated, independent colonization of extreme environments accompanied by convergent erosion of clock genes. This pattern suggests that habitat and clock gene loss are broadly associated across the phylogeny."

2c- The randomisation around line 198 doesn't take into account phylogenetic non-independence. Instead of randomising genomes, the authors should randomise branches on the tree on which losses can occur.

> The decision to re-sample genes from the genomes of the foreground was based on assessing whether circadian genes are lost more frequently than would be expected by chance given the context of the Notothenioidei or Cottioidei genome assemblies. For example, it was plausible that perhaps notothenioid genomes had more losses than other clades, resulting in an apparent increase in clock gene losses that is tied to general genome features (or assembly quality) instead of circadian biology. However, it's also a good idea to assess whether there is evidence of enrichment for circadian gene losses in other clades as well. We decided to select a set of background perciform clades to compare to Notothenioidei and Cottioiei, which showed no enrichment for circadian genes losses (Serranoidei [groupers] and Scorpaenoidei [snappers and scorpionfishes]). See above response to 2a for updated Fig. 2, methods, and results excerpts.

2d- The authors could use CAFE5 (https://doi.org/10.1093/bioinformatics/btaa1022) to evaluate significant patterns of gain and loss.

> This is a good idea to add a statical framework to our description of loss. We have added CAFE5 to our analyses, providing tables and figures to the supplemental section, and updated our methods and results section as follows:

> **Methods:**
>
> > "We used CAFE5 v1.1 (95) to test for significant contraction of biological clock gene families across Perciformes. For input into CAFE5, we generated gene count matrices with OrthoFinder v2.5.5 (90), then aggregated gene count data into four gene families: Arntl (*arntl1a*, *arntl2a*, *arntl2b*), Clock (*clocka*, *clockb*, *npas2*), Cry (*cry1a*, *cry1b*, *cry2*, *cry3a*, *cry5*, *cry-dash*), Per (*per1b*, *per2a*, *per2b*, *per3*), and Ror (*roraa*, *rorab*, *rorb*, *rorc*, *rorca*, *rorcb*)."

> **Results:**
>
> > "We detected an unexpectedly widespread pattern of clock gene loss in Perciformes, totaling 125 instances across the order (**Fig. 1**), which translated to 80 gene loss events across the Perciform phylogeny (**Fig. 2C**). These losses were concentrated in two suborders, Notothenioidei and Cottioidei, while other perciform fishes generally retained intact clock genes or had uncertain gene

statuses. At the level of gene families, *cry* and *per* paralogs were significantly contracted across the Perciform phylogeny (p=0.001 and p=0.049 respectively). The *cry* and *per* gene contractions were limited to the sub-orders Notothenioidei and Cottioidei (**Fig. S1-5; Table S3**). Contractions of *arntl/bmal*, *clock*, and *ror* gene families were insignificant."

Figures: A sample of **Fig. S5** is provided. Full supplemental materials will include a phylogeny for each gene family analyzed for Perciformes, Notothenoioidei, and Cottioidei.

Table S7 – p-values for CAFE5 analysis of gene families. Significant values (< 0.05) are bold.

Gene family	Perciformes
arntl	0.876
clock	0.893
cry	**0.001**
per	**0.049**
ror	0.965

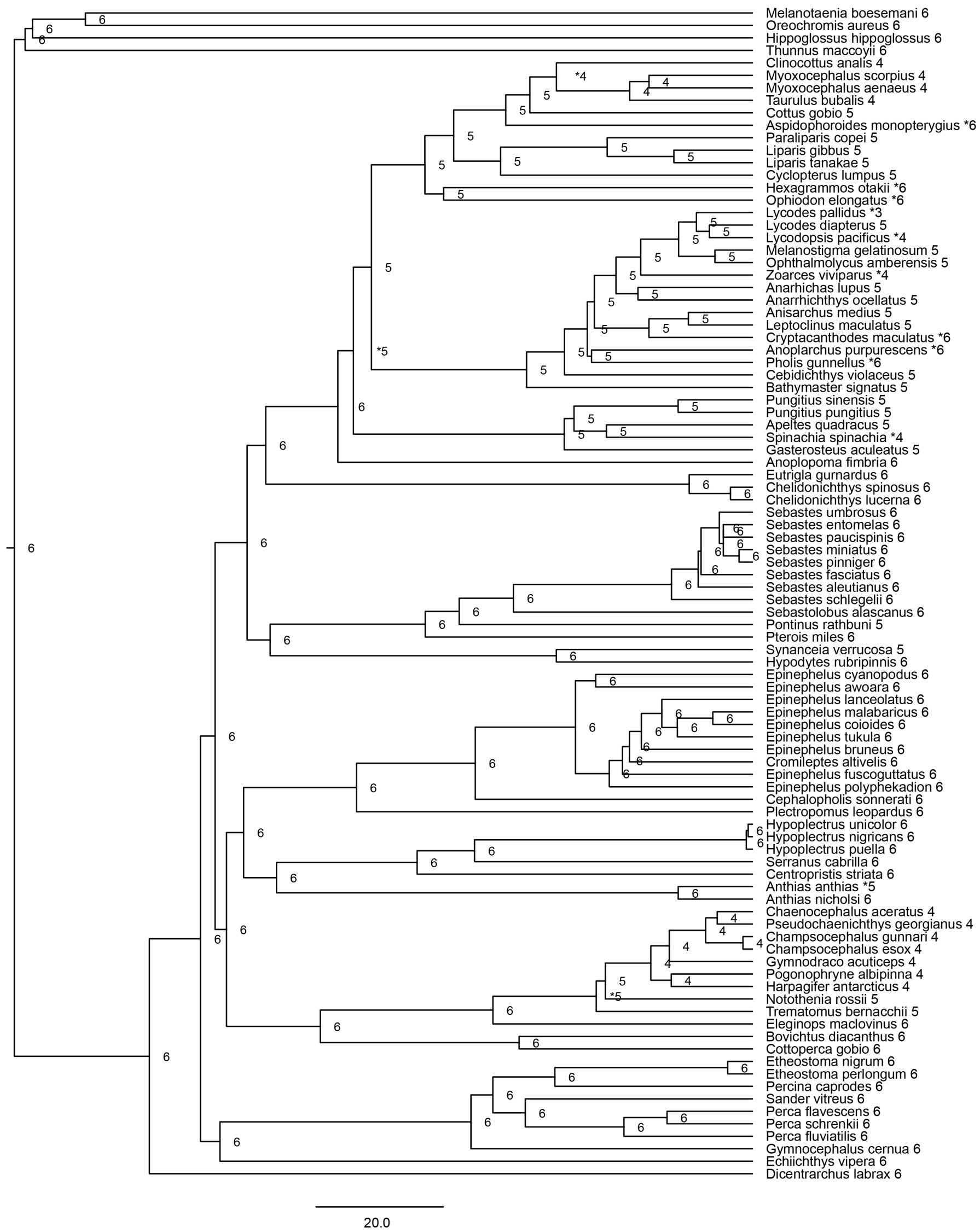

Fig. S5 – Phylogeny showing *cry* gene family for Perciformes. Nodes and tips are labeled with the inferred gene family size. Significant changes in family size from CAFE5 are indicated with an asterisk (*).

3- Potential losses of function can sometimes be solved with gene expression. For example stickleback Per1b has expression in the database Bgee (https://doi.org/10.1093/nar/gkae1118). The authors should consider checking these potential losses in expression datasets or databases.

This was an excellent suggestion and led to an important discovery regarding both our dataset and a limitation of using TOGA to annotate gene status. Of the species included in our analysis, only the three-spined stickleback is represented in Bgee. Note that we had already described *per1b* as uncertain status and not considered this as lost in our dataset. Examination of the Bgee data revealed evidence of expression for *cry2* in stickleback,

despite TOGA classifying this gene as "lost." The absence of the remaining genes was consistent with our original predictions.

To determine why TOGA failed to identify *cry2*, we used Miniprot to annotate the gene in the stickleback genome. We then performed a reciprocal BLAST analysis using the identified sequence against the reference proteome (*Sander lucioperca*), which confirmed its orthology to *cry2*. Further investigation showed that the stickleback *cry2* gene is not located within the syntenic locus occupied by *cry2* in other perciform species. As a result, it may have been missed by TOGA's genome alignment and synteny-based annotation framework.

To assess whether this issue was more widespread, we applied the same Miniprot and reciprocal BLAST pipeline to all genomes in our dataset. This analysis revealed that the problem was limited to *cry2* and affected a total of 11 species. Importantly, incorporating these newly identified *cry2* orthologs did not alter the significance of any of our results. Accordingly, the figures, methods, and results sections have been updated to reflect these changes. An updated version of Fig. 1 is reproduced below.

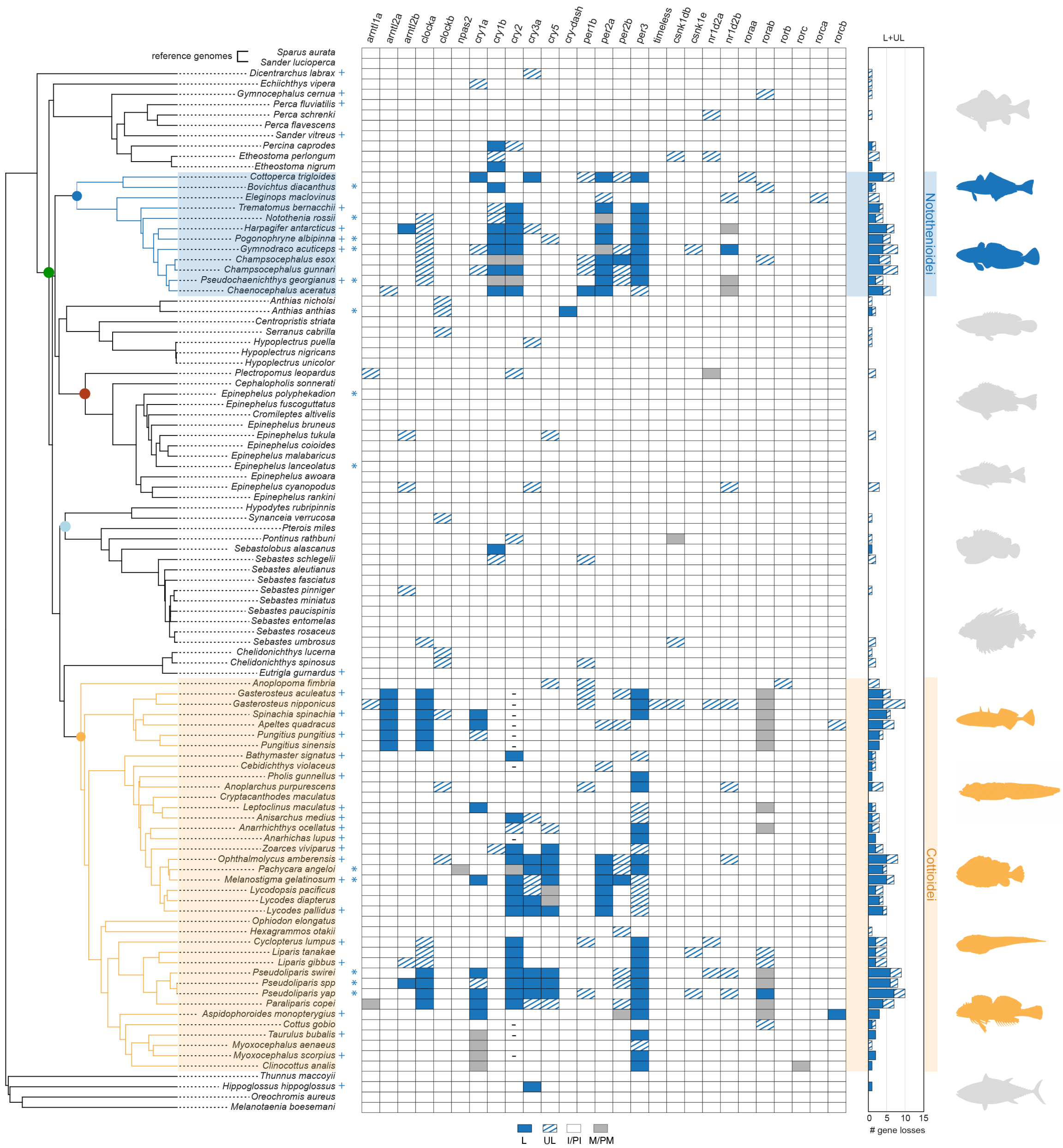

"**Fig. 1 - Loss of core biological clock genes in Perciformes.** Phylogeny of Perciformes, including five outgroup species, pruned from Rabosky et al (27). Gene status was inferred from pairwise genome alignments between each species and one of two reference genomes, *Sparus aurata* or *Sander lucioperca*. For each gene in each species, the most complete annotation across both references was used. Losses (L; blue) indicate multiple truncating mutations within the central 80% of the coding sequence (CDS). Uncertain losses (UL; blue hash) reflect a single truncating or deletion variant within the middle 80% of the gene. Gray symbols denote genes that are partially missing (PM) or part of a paralogous group (PG), suggesting possible but highly uncertain gene loss or assembly artifacts. White indicates intact or partially intact genes (PI/I; white). White squares with

dash (-) indicate instances where *cry2* was identified but out of synteny with the reference genomes. Two clades with elevated gene loss are highlighted: Notothenioidei (blue) and Cottioidei (orange). Ancestral node representing Perciformes marked with a green circle , Serranoidei with a maroon circle, and Scorpaenoidei with a light blue circle. Species whose distribution reaches polar latitudes (≥ 66°) indicated with a "+" and species whose mean depth ≥ 1000m indicated with a "*". Bar plot on the right shows the count of losses (solid blue) and uncertain losses (blue striped) for each species."

Methods:

> "To account for potential alignment, assembly, or annotation artifacts, we cross-referenced genes classified as "lost" by TOGA with the gene expression database Bgee (91) for *Gasterosteus aculeatus*, the only species shared between our dataset and the database. This comparison revealed expression of *cry2*, despite its classification by TOGA as lost in *G. aculeatus*. To identify additional misannotations across our dataset, we re-annotated all clock genes in each genome assembly using Miniprot-0.18 v1.1 (92), followed by reciprocal BLAST+ v2.14.1 (93) searches against a reference proteome. This approach recovered intact *cry2* sequences in 11 species where it was previously labeled as lost by TOGA. We therefore reclassified these genes as intact in our dataset; these corrections are indicated by dashes (–) in **Fig. 1**. These missed *cry2* annotations in TOGA likely result from the gene residing outside the expected syntenic locus in these species compared to both reference assemblies (*S. lucioperca* and *S. aurata*)."

4- The first paragraph of the Discussion is ambiguous about drift or adaptation ("likely a key component of their success"). Unless there is evidence of adaptation, the default assumption should be that these genes were lost by drift, in the absence of purifying selection maintaining their function in these environments.

We have modified the paragraph to soften our language and clarify that neutral drift is the mechanism that leads to clock gene erosion, but the widespread nature of this gene loss in suborders commonly found in high-latitude and deep-sea environments, combined with apparent flexibility of circadian metabolic rhythms in experimental data from non-polar, non-deep species suggests that a readiness to jettison a strict circadian cycle may be a part of their success in invading these habitats with limited or seasonal rhythmicity. The new paragraph is as follows:

> "We discovered that biological clock gene loss correlates with latitude and evolved convergently in the perciform suborders Notothenioidei and Cottioidei. The absence of metabolic rhythms in notothenioids links these genomic losses to a disruption in circadian systems. Together with previous studies in perciform fishes, our results

highlight an inherent circadian flexibility in both suborders. While the mechanism for gene loss is likely neutral drift under relaxed selection in high-latitude and deep-sea environments, the widespread nature of this erosion inof genes and phenotypes across each suborder suggests an ancestral release from strict circadian constraints. Reduced reliance on circadian rhythms may function as a historical contingency for each suborder that is potentially a key component of their success in high-latitude and deep-sea environments."

5- While the comparison to cave species is interesting, the authors should note that these species have evolved for much shorter times in their extreme environments than the high lattitude clades have, and with much smaller population sizes.

We have added this detail about cavefishes as recommended, though in a more general sense as the paragraph is discussing residual rhythms in a variety of contexts, and the time spent in arrhythmic environments statement could also apply to some polar and deep sea fishes as well:

"Reports of species entirely lacking circadian rhythms are rare, even in cave environments, and studies often do not exhaustively test all possible zeitgebers or potential rhythmic traits (23). For example, in the wild, the Mexican blind cavefish (*Astyanax mexicanus*) shows no molecular or behavioral circadian rhythms, but can be entrained on light-dark cycles under lab conditions (73). The Somalian cavefish (*Phreatichthys andruzzii*) has lost the ability to be entrained on artificial light dark cycles (74) despite only one reported clock gene truncation (*per2*, (75)), though remains capable of being entrained on a feeding schedule (76). There is evidence for rhythms in fish clades found well below the penetration of sunlight (1,000m) (e.g., (77, 78)), and many deep-sea invertebrates have circatidal rhythms (79)(. Within Cottioidei, an intertidal eelpout (*Zoarces viviparius*) briefly maintains circatidal swimming rhythms in captivity before transitioning to photic cues in the laboratory (80)(. Notably, these rhythms exist despite the loss of clock genes within *Z. viviparius* (**Fig.1**), indicating that many of the gene losses observed within this dataset are not sufficient to fully disrupt biological clocks. However, the retention of circadian entrainment capacity in many of these species may reflect a relatively short evolutionary timescale within arrhythmic habitats that has not yet allowed genetic drift to eliminate entrainment capacity."

6- Fig. 2 shows indentical one-sided and two-sided p-values, which is impossible. Moreover, there is no strong reason to use one-sided tests, i.e. an excess in the other direction would also have been of interest. The authors should use only two-sided tests. See e.g.

https://doi.org/10.1111/j.1442-9993.2009.01946.x, https://doi.org/10.1111/j.2041-210X.2010.00014.x.

We have removed the one-sided test as suggested and updated the methods and **Fig. 2**. See above response to comment from reviewer 1 (2a) for updated methods and figure.

7- Please provide the MCMCglmm model diagnostic plots and autocorrelation plots.

We have added model diagnostic and autocorrelation plots to supplemental materials.

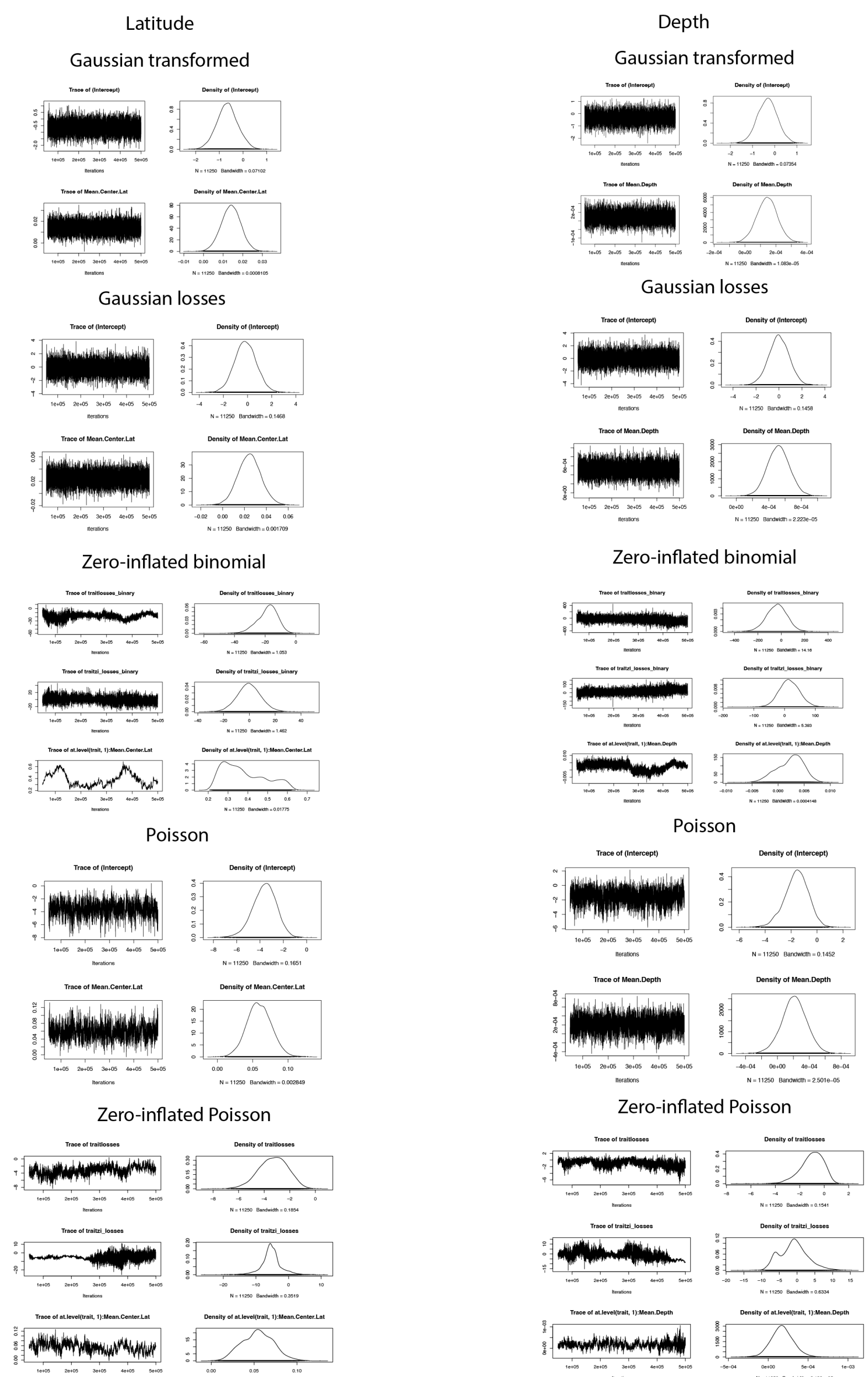

Fig. S4 – MCMCglmm trace plots. Trace plots for each of the models testing correlation between latitude (left), depth (right) and clock gene loss.

Table S5 – Autocorrelation tables for MCMCglmm models fit to latitude (left), and depth (right)

Latitude				Depth			
Gaussian transformed				Gaussian transformed			
(Intercept) Mean.Center.Lat				(Intercept) Mean.Depth			
Lag 0	1	1		Lag 0	1	1	
Lag 40	0.00141072	0.00917869		Lag 40	-0.0016407	0.01675863	
Lag 200	-0.012049	-0.0004862		Lag 200	-0.0053043	0.00794399	
Lag 400	-0.0016706	0.00217868		Lag 400	0.00576769	0.00361729	
Lag 2000	-0.0010319	0.00054751		Lag 2000	-0.0111278	0.00941804	
Gaussian losses				Gaussian losses			
(Intercept) Mean.Center.Lat				(Intercept) Mean.Depth			
Lag 0	1	1		Lag 0	1	1	
Lag 40	0.00345669	0.01859678		Lag 40	0.00168894	0.0032951	
Lag 200	-0.0048451	-0.0178741		Lag 200	0.00092289	0.0102586	
Lag 400	0.00683902	0.00322361		Lag 400	0.01342535	-0.0109099	
Lag 2000	-0.0143491	-0.0075259		Lag 2000	0.00258877	0.00835541	
Zero-inflated binomial				Zero-inflated binomial			
(trait, 1):Mean.Center.Lat				(trait, 1):Mean.Depth			
Lag 0	1	1	1	Lag 0	1	1	1
Lag 40	0.7854068	0.1457199	0.931426	Lag 40	0.1275081	0.1403428	0.64959
Lag 200	0.7778938	0.1728338	0.9247226	Lag 200	0.1427242	0.1558557	0.6410716
Lag 400	0.7682594	0.1519089	0.9170385	Lag 400	0.1297132	0.1421099	0.6402735
Lag 2000	0.7025019	0.1386631	0.8703137	Lag 2000	0.1374242	0.1504865	0.6355919
Poisson				Poisson			
(Intercept) Mean.Center.Lat				(Intercept) Mean.Depth			
Lag 0	1	1		Lag 0	1	1	
Lag 40	0.6151098	0.71202307		Lag 40	0.26689495	0.29243909	
Lag 200	0.4821211	0.48006662		Lag 200	0.22677482	0.12044293	
Lag 400	0.3734059	0.31656096		Lag 400	0.18342931	0.06168543	
Lag 2000	0.1114785	0.04976782		Lag 2000	0.08745207	0.00723057	
Zero-inflated Poisson				Zero-inflated Poisson			
(trait, 1):Mean.Center.Lat				(trait, 1):Mean.Depth			
Lag 0	1	1	1	Lag 0	1	1	1
Lag 40	0.7246228	0.3960429	0.972894	Lag 40	0.5077879	0.5683023	0.7277525
Lag 200	0.697535	0.3813361	0.9420032	Lag 200	0.4881581	0.5534513	0.6119857
Lag 400	0.6887421	0.3954298	0.9094095	Lag 400	0.4724555	0.5498725	0.5162124
Lag 2000	0.549636	0.3276456	0.7236152	Lag 2000	0.4028465	0.4995034	0.2233435

8a- In Table S2, please provide BUSCO or equivalent scores for the genomes, which is important for this type of study of gene loss.

8b- Statistics are missing for H. otakii.

BUSCO data and the data for H. otakii has been added to **Table S2**:

Order	Suborder	Family	Species	GenBank Accession	Length (Mb)	Scaffold count	Scaffold N50 (Mb)	Contig count	Contig N50 (Mb)	BUSCO C:
Perciformes	Cottioidei	Anarhichadidae	Anarhichas lupus	GCA_050578055.1	627.7	178	23.8	178	23.8	98.6%
Perciformes	Cottioidei	Anarhichadidae	Anarrhichthys ocellatus	GCF_004355925.1	612.8	10816	5.7	33924	41.8kb	94.9%
Perciformes	Cottioidei	Stichaeidae	Anisarchus medius	GCA_046127435.1	669.3	23	30.2	285	11.0	97.5%
Perciformes	Cottioidei	Stichaeidae	Anoplarchus purpurescens	GCA_041002825.1	560.8	486	10.7	492	10.4	98.2%
Perciformes	Cottioidei	Anoplopomatidae	Anoplopoma fimbria	GCA_027596085.2	653.5	7493	26.7	8212	2.6	98.3%
Perciformes	Serranoidei	Anthiadidae	Anthias anthias	GCA_964269795.2	796.1	65	34.7	168	17.3	97.8%
Perciformes	Serranoidei	Anthiadidae	Anthias nicholsi	GCA_051175935.1	800.3	264	14.7	264	14.7	98.4%
Perciformes	Cottioidei	Gasterosteidae	Apeltes quadracus	GCA_048569185.1	475.9	215	18.5	339	10.5	97.7%
Perciformes	Cottioidei	Agonidae	Aspidophoroides monopterygius	GCA_051200695.1	658.0	703	7.4	703	7.4	95.8%
Perciformes	Cottioidei	Bathymasteridae	Bathymaster signatus	GCA_050575475.1	712.3	1431	3.1	1431	3.1	98.3%
Perciformes	Notothenioidei	Bovichtidae	Bovichtus diacanthus	GCA_943590825.1	641.8	12443	7.3	44874	0.0	92.9%
Perciformes	Cottioidei	Stichaeidae	Cebidichthys violaceus	GCA_008087265.1	593.0	467	6.7	505	5.5	93.4%
Perciformes	Serranoidei	Serranidae	Centropristis striata	GCA_030273125.1	926.0	92	39.0	433	9.5	98.7%
Perciformes	Serranoidei	Serranidae	Cephalopholis sonnerati	GCA_043388425.1	1039.5	24	43.8	24	43.8	98.9%
Perciformes	Notothenioidei	Channichthyidae	Chaenocephalus aceratus	GCA_023974075.1	1065.6	3134	33.5	3856	1.5	93.0%
Perciformes	Notothenioidei	Channichthyidae	Champsocephalus esox	GCA_036324585.1	987.1	2069	43.6	3502	2.6	97.6%
Perciformes	Notothenioidei	Channichthyidae	Champsocephalus gunnari	GCA_036324595.1	994.2	1536	44.1	2993	3.2	98.0%
Perciformes	Triglioidei	Triglidae	Chelidonichthys lucerna	GCA_965644425.1	651.6	345	27.9	650	3.4	98.6%
Perciformes	Triglioidei	Triglidae	Chelidonichthys spinosus	GCA_029853015.1	624.7	293	28.1	406	13.8	98.1%
Perciformes	Cottioidei	Cottidae	Clinocottus analis	GCA_023055335.1	538.1	443	21.0	662	9.2	97.9%
Perciformes	Notothenioidei	Bovichtidae	Cottoperca trigloides	GCA_900634415.1	609.4	322	25.2	766	6.3	94.0%
Perciformes	Cottioidei	Cottidae	Cottus gobio	GCA_023566465.1	645.6	161	28.0	309	10.5	98.4%
Perciformes	Serranoidei	Serranidae	Cromileptes altivelis	GCA_013133815.1	1013.4	164	43.5	283	18.3	97.0%
Perciformes	Cottioidei	Cryptacanthodidae	Cryptacanthodes maculatus	GCA_040955035.1	733.7	501	5.5	501	5.5	98.2%
Perciformes	Cottioidei	Cyclopteridae	Cyclopterus lumpus	GCA_009769545.1	572.9	49	23.9	396	5.0	97.0%
Acanthuriformes	N/A	Moronidae	Dicentrarchus labrax	GCA_905237075.1	695.9	302	29.9	574	12.7	98.9%
Perciformes	Percoidei	Trachinidae	Echiichthys vipera	GCA_963691815.1	800.4	133	33.0	739	2.6	98.2%
Perciformes	Notothenioidei	Eleginopidae	Eleginops maclovinus	GCA_036324505.1	606.3	26	26.7	406	7.6	98.1%
Perciformes	Serranoidei	Serranidae	Epinephelus awoara	GCA_035609425.1	984.5	49	41.4	65	39.8	98.8%
Perciformes	Serranoidei	Serranidae	Epinephelus bruneus	GCA_041684075.1	1079.6	863	44.3	1184	37.8	98.3%
Perciformes	Serranoidei	Serranidae	Epinephelus coioides	GCA_051314025.1	1071.9	33	45.6	140	18.7	98.9%
Perciformes	Serranoidei	Serranidae	Epinephelus cyanopodus	GCA_026686955.1	998.8	106	42.0	458	5.9	98.2%
Perciformes	Serranoidei	Serranidae	Epinephelus fuscoguttatus	GCF_011397635.1	1047.0	26	44.4	231	13.9	98.8%
Perciformes	Serranoidei	Serranidae	Epinephelus lanceolatus	GCA_041903045.1	1089.4	176	45.8	183	44.4	98.3%
Perciformes	Serranoidei	Serranidae	Epinephelus malabaricus	GCA_037127435.1	1107.1	423	43.3	790	4.7	98.6%
Perciformes	Serranoidei	Serranidae	Epinephelus polyphekadion	GCA_046464035.1	1079.2	74	45.9	263	28.1	99.0%
Perciformes	Serranoidei	Serranidae	Epinephelus rankini	GCA_048129065.1	1019.0	199	42.1	232	34.9	98.9%
Perciformes	Serranoidei	Serranidae	Epinephelus tukula	GCA_021130775.1	1130.5	1557	44.7	2268	5.4	98.8%
Perciformes	Serranoidei	Percidae	Etheostoma nigrum	GCA_029931835.1	764.0	3560	30.2	6012	0.8	97.2%
Perciformes	Percoidei	Percidae	Etheostoma perlongum	GCA_026937815.1	788.7	1179	30.8	2095	2.3	97.0%
Perciformes	Triglioidei	Triglidae	Eutrigla gurnardus	GCA_963514095.1	680.5	319	29.2	872	2.4	98.4%
Perciformes	Cottioidei	Gasterosteidae	Gasterosteus aculeatus	GCA_016920845.1	471.9	2937	20.5	6059	0.5	97.2%
Perciformes	Cottioidei	Gasterosteidae	Gasterosteus nipponicus	GCA_014132575.2	599.5	3095	17.6	3718	0.5	96.8%
Perciformes	Percoidei	Percidae	Gymnocephalus cernua	GCA_023631565.1	904.3	156	38.2	583	10.9	98.1%
Perciformes	Notothenioidei	Bathydraconidae	Gymnodraco acuticeps	GCA_902827175.1	996.9	2618	1.9	4183	0.5	93.8%
Perciformes	Notothenioidei	Harpagiferidae	Harpagifer antarcticus	GCA_902827135.1	941.5	1541	5.0	2579	1.1	94.4%
Perciformes	Cottioidei	Hexagrammidae	Hexagrammos otakii	GCA_043790635.1	682.4	493	27.8	1380	2.4	97.7%
Pleuronectiformes	N/A	Pleuronectidae	Hippoglossus hippoglossus	GCA_009819705.1	596.8	56	26.3	346	7.0	98.6%
Perciformes	Scorpaenoidei	Tetrarogidae	Hypodytes rubripinnis	GCA_043172655.1	919.8	421	4.9	426	4.8	97.8%
Perciformes	Serranoidei	Serranidae	Hypoplectrus nigricans	GCA_964304555.1	685.2	315	26.0	334	25.0	98.7%
Perciformes	Serranoidei	Serranidae	Hypoplectrus puella	GCA_964304535.1	686.8	253	26.1	268	23.2	97.5%
Perciformes	Serranoidei	Serranidae	Hypoplectrus unicolor	GCA_964304545.1	692.8	285	26.8	295	25.3	98.6%
Perciformes	Cottioidei	Stichaeidae	Leptoclinus maculatus	GCA_032191485.1	699.9	586	23.5	1079	2.4	98.1%
Perciformes	Cottioidei	Liparidae	Liparis gibbus	GCA_040955725.1	677.8	5180	0.6	5180	0.6	94.8%
Perciformes	Cottioidei	Liparidae	Liparis tanakae	GCA_043790635.1	682.4	493	27.8	1380	2.4	97.2%
Perciformes	Cottioidei	Zoarcidae	Lycodes diapterus	GCA_050575495.1	711.9	677	6.0	677	6.0	98.6%
Perciformes	Cottioidei	Zoarcidae	Lycodes pallidus	GCA_049309625.1	753.3	627	28.6	988	5.0	98.9%
Perciformes	Cottioidei	Zoarcidae	Lycodopsis pacificus	GCA_028022725.1	646.4	109	27.7	475	4.7	98.2%
Perciformes	Cottioidei	Zoarcidae	Melanostigma gelatinosum	GCA_949748355.1	662.0	534	28.0	1123	10.5	98.1%
Atheriniformes	N/A	Melanotaeniidae	Melanotaenia boesemani	GCA_017639745.1	865.6	92	37.9	532	9.3	98.8%
Perciformes	Cottioidei	Cottidae	Myoxocephalus aenaeus	GCA_038501785.1	719.9	1019	4.5	1019	4.5	98.2%
Perciformes	Cottioidei	Cottidae	Myoxocephalus scorpius	fMyoSco1	720.0	98.0	36.4	585	3.1	98.5%
Perciformes	Notothenioidei	Nototheniidae	Notothenia rossii	GCA_949606895.1	1042.9	943	89.7	5100	0.4	94.4%
Perciformes	Cottioidei	Hexagrammidae	Ophiodon elongatus	GCA_016806645.1	645.9	2175	1.7	2224	1.5	97.4%
Perciformes	Cottioidei	Zoarcidae	Ophthalmolycus amberensis	GCA_024529925.1	680.7	1825	1.0	1825	1.0	98.0%
Cichliformes	N/A	Cichlidae	Oreochromis aureus	GCA_013358895.1	1000.0	303	40.7	1025	4.2	98.6%
Perciformes	Cottioidei	Zoarcidae	Pachycara angeloi	GCA_048544875.1	618.2	1573	26.8	2878	25.3	96.4%
Perciformes	Cottioidei	Liparidae	Paraliparis copei	GCA_051313365.1	627.7	2333	0.8	2333	0.8	95.1%
Perciformes	Percoidei	Percidae	Perca flavescens	GCA_004354835.1	877.4	267	37.4	1096	4.3	98.7%
Perciformes	Percoidei	Percidae	Perca fluviatilis	GCF_010015445.1	951.3	303	39.6	1092	4.2	97.7%
Perciformes	Percoidei	Percidae	Perca schrenkii	GCA_025617405.1	908.1	1258	36.4	2019	3.2	98.3%
Perciformes	Percoidei	Percidae	Percina caprodes	GCA_013375575.1	1012.0	3656	1.4	3658	1.4	97.8%
Perciformes	Cottioidei	Pholidae	Pholis gunnellus	GCA_910591455.2	588.7	101	25.4	519	3.5	98.7%
Perciformes	Serranoidei	Serranidae	Plectropomus leopardus	GCA_026936395.1	849.8	120	38.0	168	33.2	94.8%
Perciformes	Notothenioidei	Artedidraconidae	Pogonophryne albipinna	GCA_028583405.1	1074.5	1111	41.8	3897	1.0	97.1%
Perciformes	Scorpaenoidei	Scorpaenidae	Pontinus rathbuni	GCA_047676105.1	928.7	453	9.8	453	9.8	98.6%
Perciformes	Notothenioidei	Channichthyidae	Pseudochaenichthys georgianus	GCA_902827115.2	1026.2	1562	42.8	4129	0.7	94.4%
Perciformes	Cottioidei	Liparidae	Pseudoliparis spp	GCA_048564765.1	802.8	135	33.2	778	5.3	96.4%
Perciformes	Cottioidei	Liparidae	Pseudoliparis swirei	GCA_029220125.1	626.4	199	25.7	1102	4.2	96.2%
Perciformes	Cottioidei	Liparidae	Pseudoliparis yap	GCA_004335475.1	840.6	3134	1.0	6333	1.0	92.7%
Perciformes	Scorpaenoidei	Scorpaenidae	Pterois miles	GCA_947000775.1	902.4	N/A	N/A	660	14.5	98.6%
Perciformes	Cottioidei	Gasterosteidae	Pungitius pungitius	GCA_949316345.1	480.5	175	21.0	913	1.4	97.9%
Perciformes	Cottioidei	Gasterosteidae	Pungitius sinensis	GCA_049949165.1	479.7	21	22.4	38	21.9	98.1%
Perciformes	Percoidei	Percidae	Sander lucioperca	GCA_008315115.1	900.5	1312	4.9	1347	4.7	98.5%
Perciformes	Percoidei	Percidae	Sander vitreus	GCA_031162955.1	791.7	684	33.3	1003	6.2	98.4%
Perciformes	Scorpaenoidei	Sebastidae	Sebastes aleutianus	GCA_916700875.1	889.5	726	34.1	1168	5.8	98.4%
Perciformes	Scorpaenoidei	Sebastidae	Sebastes entomelas	GCA_045838235.1	819.6	76	35.4	216	18.9	98.9%
Perciformes	Scorpaenoidei	Sebastidae	Sebastes fasciatus	GCA_047301235.2	840.9	126	36.1	408	18.7	98.7%
Perciformes	Scorpaenoidei	Sebastidae	Sebastes miniatus	GCA_916701275.1	850.7	568	33.1	837	8.6	98.7%
Perciformes	Scorpaenoidei	Sebastidae	Sebastes paucispinis	GCA_036937225.1	806.9	211	34.6	378	14.3	98.8%
Perciformes	Scorpaenoidei	Sebastidae	Sebastes pinniger	GCA_916701065.2	825.8	606	10.1	606	10.1	98.2%
Perciformes	Scorpaenoidei	Sebastidae	Sebastes rosaceus	GCA_916701555.1	959.6	881	31.7	1407	3.0	98.1%
Perciformes	Scorpaenoidei	Sebastidae	Sebastes schlegelii	GCA_014673565.1	848.0	1663	7.9	2110	5.5	98.2%
Perciformes	Scorpaenoidei	Sebastidae	Sebastes umbrosus	GCF_015220745.1	800.9	138	34.9	353	11.4	98.3%
Perciformes	Scorpaenoidei	Sebastidae	Sebastolobus alascanus	GCA_916701645.2	889.8	3395	2.0	3395	2.0	98.5%
Perciformes	Scorpaenoidei	Serranidae	Serranus cabrilla	GCA_965643205.1	735.1	220	30.0	223	30.0	98.9%
Spariformes	N/A	Sparidae	Sparus aurata	GCA_900880675.1	833.2	175	35.8	1223	2.9	98.4%
Perciformes	Cottioidei	Gasterosteidae	Spinachia spinachia	GCA_048126635.1	407.6	38	19.4	279	6.6	98.0%
Perciformes	Scorpaenoidei	Synanceiidae	Synanceia verrucosa	GCA_029721515.1	691.9	1532	27.1	2325	12.0	97.9%
Perciformes	Cottioidei	Cottidae	Taurulus bubalis	GCA_910589615.1	615.2	27	29.1	269	13.0	98.6%
Scombriformes	N/A	Scombridae	Thunnus maccoyii	GCA_910596095.1	782.4	57	33.8	152	26.8	99.2%
Perciformes	Notothenioidei	Nototheniidae	Trematomus bernacchii	GCA_902827165.1	867.1	864	8.8	1793	1.4	97.6%
Perciformes	Cottioidei	Zoarcidae	Zoarces viviparus	GCA_040110945.1	663.0	607	26.1	817	10.9	98.5%

Minor comments:
9- It is worth noting that Ref 42 Toloza-Villalobos et al which is discussed several times did not consider all of the genes which are studied here, e.g. they don't consider Bmal.

This is an important point and we have updated the text to clarify that not all genes included in our study were part of prior studies reference in the manuscript.

> "Of the 33 candidate clock genes, 26 genes were annotated in both reference genomes (S. lucioperca, S. aurata). Of the seven genes not annotated in these references, one gene, rorab (S. lucioperca: ENSSLUG00000016495; S. aurata: ENSSAUG00010006455), was identified as a previously unannotated gene using a de novo annotation approach with Exonerate (61). For the remaining six genes (arntl1b, cry3b, cry4, per1a, csnk1da, and nr1d1), no orthologs were detected with either TOGA or Exonerate in either reference genome; thus, they are presumed to be absent from the genomes of all perciform species included in the study. The absence of these genes is consistent with observed patterns for other members of Acanthomorpha, though previous studies did not consider all genes included here (42)."

10- It would be informative to show transitions to high lattitude or deep sea on the branches of trees of figs. 1 and 4.

This is a good suggestion and helpful information to include in the figures. We indicated deep-sea and high latitude transitions with symbols (+ = high-latitude, * = deep-sea) in the original version of Fig. 1 as we felt it was easily interpreted and made it clear which species lived in both high-latitude, and deep-sea environments. For Fig. 4, we have mapped habitat as a trait with stochastic character mapping and mirrored the results with the gene loss mapping to visually depict the relationship between clock gene loss and extreme habitats. See responses above for the updated figures and text excerpts.

Reviewer 2:
1. The paper would benefit from greater discussion the role of clock genes outside of Zeitgeber regulation (non-circadian). Many have functions that differ from their role in in daily rhythms. Presumably loss of clock-genes has wide-ranging effects on physiological and cellular function.

The reviewer is correct that clock genes have roles outside of zeitgeber regulation. To address this, we have augmented our discussion of what is known about clock gene function outside of maintenance of the circadian clock.

> "Our data indicate distinct preservation patterns in core biological clock gene paralogs within Perciformes, potentially highlighting reliance on specific clock genes over others (**Fig. 2C-E**). Many infrequently lost genes have pleiotropic functions outside the clock. For example, Cry5, Cry-dash, and Timeless contribute to DNA repair, and retinoid-related orphan receptors (Ror) are involved in immune, metabolic, and developmental processes (52, 56, 64). These broader functions likely explain their retention in Perciformes. The consistent presence of *clockb*,

npas2, and *arntl1a* also suggests essential roles. In contrast, the species-specific retention of either *cry1a* or *cry1b* suggests some functional redundancy between these paralogs. Retention of at least one paralog could be related to residual circadian clock functions or to other essential roles of Cry1 proteins, such as DNA repair (65). One of the most consistent losses is *per3*, which is absent in multiple species of Notothenioidei and Cottioidei. This gene is frequently lost across teleosts (42), including in Atlantic cod (*Gadus morhua*), northern pike (*Esox lucius*), three-spined stickleback (48), and in many salmonids (49, 66), suggesting a diminished role in fish circadian regulation. Still, *per3* remains under intensified purifying selection in Atlantic salmon (*S. salar*), and brown trout (*Salmo trutta*) (49, 66). In contrast, *per1b* and at least one copy of *per2* are relatively well conserved across teleosts (49), including in our dataset (**Fig. 1**)."

2. Why would a species lose seven circadian genes? Are there shared or partial functions? Presumably loss of one, or a few, key genes can fully disable the clock. If there is a way to extend the analysis in 2E,F to discuss the broader functions of the circadian clock? Could the analysis be extended to compare core-clock genes vs output-associated genes?

This is an excellent question. We argue that once there is relaxed selection on the clock, genes under neutral drift will randomly accumulate mutations. This would result in multiple family members having truncating variants. As to why some species have more losses than others, this may just be reflection of evolutionary time with clock phenotypes under relaxed selection or perhaps due to differences in mutation rate or other parameters (e.g., effective population size)

As noted in the manuscript, studies suggest that more than 40% of genes may be under some measure of circadian regulation. If circadian rhythms are indeed functionally lost, there would be numerous downstream impacts to clock-controlled gene expression. While an interesting topic, this is a question for future study and outside the scope of the current manuscript. We have updated the summary calling for future research on this topic.

"We find that many core biological clock genes are disrupted in two perciform clades common to high-latitude and deep-sea environments: Notothenioidei and Cottioidei. The extent of gene loss correlates with latitude, with polar species showing a higher extent of loss than temperate lineages. We also show that both Antarctic and sub-Antarctic notothenioids lack daily metabolic rhythms, instead exhibiting steady-state baseline metabolism like that observed in Cottioidei under constant lighting. While high-latitude and deep-sea habitats impose diverse selective pressures, our findings support the idea that altered circadian regulation (through plasticity and genetic erosion) represents a release from an ancestral constraint on strict circadian adherence, potentially fueling their success within arrhythmic habitats. Our study focuses on genes associated with the clock mechanism, but it remains unknown how downstream pathways react to a loss of clock genes, a topic for future research. Because chronic circadian disruption is deleterious in many vertebrates, uncovering how these fishes maintain physiological stability despite reduced circadian control may offer insights into the mechanisms that support resilience in arrhythmic environments."

3. Some reorganization and clarification of jargon would make this more accessible to a generalist audience. For example, see paragraph at line 225.

> Accessibility of the material is always a concern. We removed or clarified jargon wherever possible in the manuscript and specifically addressed the paragraph at 225:
>
> > "Given the observed patterns of gene loss, we hypothesized that biological clock genes were lost independently in Notothenioidei and Cottioidei. To test this hypothesis, we used stochastic character mapping to estimate the probability of clock gene loss in ancestral nodes of the Perciform phylogeny given the observed losses at the tips (**Fig. 4**). Clock loss was assigned if tip species had one or more losses (≥ 1 loss) in the panel of 27 analyzed genes. To provide a comparison to transitions into deep-sea or high-latitude environments, we also mapped the extreme ranges of species habitat onto the phylogeny (**Fig. 4)**."

4. Could the authors discuss on changes in circadian genes in highly divergent subterranean animals with loss of circadian genes including mole rates and cavefish?

> Cavefish are an excellent model for circadian loss, though there is more evidence for altered circadian rhythms than circadian gene status. While briefly mentioned in the original manuscript, we have elaborated on what is known of their circadian gene loss and function:
>
> > "Reports of species entirely lacking circadian rhythms are rare, even in cave environments, and studies often do not exhaustively test all possible zeitgebers or potential rhythmic traits (23). For example, in the wild, the Mexican blind cavefish (Astyanax mexicanus) shows no molecular or behavioral circadian rhythms, but can be entrained on light-dark cycles under lab conditions (73). The Somalian cavefish (Phreatichthys andruzzii) has lost the ability to be entrained on artificial light dark cycles (74) despite only one reported clock gene truncation (per2, (75)), though remains capable of being entrained on a feeding schedule (76). There is evidence for rhythms in fish clades found well below the penetration of sunlight (1,000m) (e.g., (77, 78)(73, 74)), and many deep-sea invertebrates have circatidal rhythms (79)(75). Within Cottioidei, an intertidal eelpout (*Zoarces viviparius*) briefly maintains circatidal swimming rhythms in captivity before transitioning to photic cues in the laboratory (80)(76). Notably, these rhythms exist despite the loss of circadian clock genes within *Z. viviparius* (**Fig.1**), indicating that many of the gene losses observed within this dataset are not sufficient to fully disrupt biological clocks. However, the retention of circadian entrainment capacity in many of these species may reflect a relatively short evolutionary timescale within arrhythmic habitats that has not yet allowed genetic drift to eliminate entrainment capacity"

5. It would be helpful to discuss why loss of clock genes might be advantageous, and particularly how the loss of specific Zeitgebers may push adaptive pressure towards loss of the clock.

We agree that further elaboration is necessary to clarify how the loss of clock genes could be advantageous. We have added the following to our discussion section on "Clocks and survival in high latitudes and the deep sea":

> "Fish clades with the fastest speciation rates often include species found in both polar and deep-sea environments, suggesting that there may be underlying shared traits that enable survival in both of these extreme habitats (29). While most studies focus on cold temperatures or high hydrostatic pressures in these environments, the breakdown of environmental zeitgebers and their impact on engrained circadian mechanisms is also likely an adaptive barrier (25). Further, in seasonally or permanently arrhythmic environments, responding opportunistically, rather than anticipating cyclic cues, may be advantageous in order to exploit unpredictable conditions. Non-polar and non-deep perciforms show signs of diel flexibility. In addition to the stickleback example mentioned above (39), the slimy sculpin (*Cottus cognatus*; Cottioidei) in Lake Ontario (lat 43°N) shows evidence of diel rhythms in feeding as juveniles at higher water depths (35 m) but has arrhythmic feeding patterns as adults at 75 m (85). In our dataset, the sub-Antarctic notothenioid *E. maclovinus* had no clock gene losses and only three uncertain losses but shows the same lack of metabolic oscillation as *P. georgianus* and *C. aceratus* (**Figs. 1**, **6**). The absence of detectable metabolic rhythmicity in *E. maclovinus*, despite its largely intact gene complement, suggests that phenotypic loss of some circadian functions may have predated the extensive genomic erosion observed in Antarctic lineages. As evidenced by the absence or seasonal plasticity of metabolic rhythms combined with widespread gene losses across multiple perciform species (39–41), this circadian flexibility is an underappreciated component of the invasion of notothenioid and other perciforms into environmental extremes."

Reviewer 3:

1) Clock gene redundancy: the manuscript briefly touches on redundancy of clock gene paralogs, but I think this needs to be expanded upon given the limited functional support. In many other systems, clock paralogs are lost while rhythms are maintained, and knockdownsin mice demonstrate that rhythms can be maintained even when individual clock genes areabsent due to genetic redundancy. Looking at Figure 1, it seems that there are very few cases where multiple paralogs in the core clock show losses in the same species — for instance, while cry1b is lost in several notothenioids, cry1a appears retained, and no species appears to have lost both paralogs simultaneously. The authors should more explicitly address whether the observed single-paralog losses are likely to be functionally disruptive given what is known about clock gene redundancy.

> This is an important observation that we do make in the manuscript. Regardless, the role paralogs play is worthy of greater discussion. To address the reviewer's comment, especially as they pertain to *cry1* paralogs, we have added to our discussion of the role of paralogs based on what is known about their function.

"Our data indicate distinct preservation patterns in core biological clock gene paralogs within Perciformes, potentially highlighting reliance on specific clock genes over others (**Fig. 2C-E**). Many infrequently lost genes have pleiotropic functions outside the clock. For example, Cry5, Cry-dash, and Timeless contribute to DNA repair, and retinoid-related orphan receptors (Ror) are involved in immune, metabolic, and developmental processes (52, 56, 64). These broader functions likely explain their retention in Perciformes. The consistent presence of *clockb*, *npas2*, and *arntl1a* also suggests essential roles. In contrast, the species-specific retention of either *cry1a* or *cry1b* suggests some functional redundancy between these paralogs. Retention of at least one paralog could be related to residual circadian clock functions or to other essential roles of Cry1 proteins, such as DNA repair (65). One of the most consistent losses is *per3*, which is absent in multiple species of Notothenioidei and Cottioidei. This gene is frequently lost across teleosts (42), including in Atlantic cod (*Gadus morhua*), northern pike (*Esox lucius*), three-spined stickleback (48), and in many salmonids (49, 66), suggesting a diminished role in fish circadian regulation. Still, *per3* remains under intensified purifying selection in Atlantic salmon (*S. salar*), and brown trout (*Salmo trutta*) (49, 66). In contrast, *per1b* and at least one copy of *per2* are relatively well conserved across teleosts (49), including in our dataset (**Fig. 1**).."

2) Further delineation of the "uncertain losses" category would provide important biological context for Figure 1. The current presentation groups several distinct situations (fragmented assemblies, single truncating mutations, etc) under a single "uncertain" designation. Breaking these down to report uncertain cases by more specific designations would help with interpretation.

This is a great idea. We have provided a new Table S4, a snippet of which is reproduced below showing data for two example uncertain losses (the entire table is a 2800 line spreadsheet). This table allows us to present mutation data for each gene and species. This table lists the truncating variants per gene, exon, and species based on the longest isoform. The mutations are sorted by masked (which typically indicates does not cause a frameshift) or unmasked, and contains the overall percent intact reading frame for the transcript in question.

Table S4 - Mutation description for genes with status uncertain loss (UL).

Gene_ID	Species	Best_Transcript_ID	Percent_Intact	Masked_Variants	Unmasked_Variants
ENSSLUG00000010714	anoplopoma_fimbria	ENSSAUT00010035032	65%	NA	exon 15/19:FS_DEL(-1)
ENSSLUG00000015034	anoplopoma_fimbria	ENSSAUT00010039340	89%	NA	exon 1/10:FS_DEL(-1)

3) Tests for selection: The authors suggest that losses may reflect relaxed selection on clock genes in arrhythmic environments. Can the authors examine this question by explicitly testing whether there is evidence for relaxed selection on clock genes?

The reviewer is correct that it is possible to explicitly test for relaxed selection. We performed an analysis on relaxed selection using the program RELAX, however, we found that the analysis was highly sensitive to the transcript selected (longest transcript vs a gene-tree informed approach such as Isosel) and the reference genome used (*Sander lucioperca*, *Sparus aurata*). Depending on the transcript selection and reference, we would see variation in not only the p-value for genes, but the direction of selection (significantly relaxed vs. significantly intensified, see examples in table below).

Sander lucioperca	common name	K species	p.adj species	status species	Sparus aurata	K species	p.adj species	status species
ENSSLUG00000012954	cry3a	20.59191717	0.000436193	intensification	ENSSAUG00010004169	0.709227053	4.43E-08	relaxation
ENSSLUG00000016495	rora	0.719372595	0.001866053	relaxation	ENSSAUG00010006455	2.165626321	0.013073481	intensification
ENSSLUG00000011504	per3	0.308770772	5.39E-05	relaxation	ENSSAUG00010011519	12.53516789	9.84E-11	intensification
ENSSLUG00000023039	arntl2b	3.313159587	0.002751536	intensification	ENSSAUG00010013586	0.636311915	0.036351262	relaxation

When we limited the analysis to genes with consistent results regardless of transcript selection and reference genome, there were 2 genes with significantly relaxed selection that did not already have gene losses (a clearer indicator of relaxed selection). Given how little the analysis contributed to the main findings of the paper and our misgivings about the reliability of the results, we chose not to include it in the manuscript.

4) The authors suggest that the loss of rhythms potentially facilitated the repeated expansion of these fishes into polar and deep-sea habitats. I am not sure this argument is well supported, as it relies on observations of E. maclovinus alone. I would think the loss of clock genes could also reflect drift after movement into environments with reduced rhythmicity, particularly given the association observed with latitude. E. maclovinus also seems unusual case for driving this argument, as this species has no circadian gene losses as observed in the other species and shows the same oscillation patterns as species with substantial gene loss. This raises questions as to what is driving the apparent metabolic arrhythmia and whether this instead reflects the experimental conditions rather than evolved differences in clock function.

We agree with the reviewer that drawing definitive conclusions from the E. maclovinus data alone requires caution. Accordingly, we have added tempering language to the Discussion to clarify these limitations (please see our response to Reviewer 1, Comment 4). Importantly, our original interpretation was also informed by published work in sculpins and three-spined stickleback. These studies demonstrate that individuals from regions with

regular photoperiods still exhibit natural variation in metabolic rhythmicity and/or flexible seasonal shifts in rhythmicity. We hypothesize that this inherent flexibility (the ability to tolerate disrupted rhythms without obvious fitness trade-offs) initially facilitates expansion into aphotic environments. Once a lineage colonizes an arrhythmic environment, subsequent relaxed selection drives the erosion of the core clock genes observed in our dataset.

> "The absence of detectable metabolic rhythmicity in *E. maclovinus,* despite its largely intact gene complement, suggests that phenotypic loss of circadian functions may have predated the extensive genomic erosion observed in Antarctic lineages. As evidenced by the absence or seasonal plasticity of metabolic rhythms combined with widespread gene losses across multiple perciform species (39–41), this circadian flexibility is an underappreciated component of the invasion of notothenioid and other perciforms into environmental extremes."

Reviewer 4:
The figures 1-4 seem to have somewhat redundant information. The information is displayed nicely but it's unclear to me how different the information is that is being displayed in each figure.

It is true that the underlying data has some overlap by being based on patterns of gene loss, we believe the plots show the data in ways that would be difficult within one figure and merits separate display items. For example, Fig. 1 shows an overview of the entire dataset allowing the reader to match specific gene loss predictions to specific species while also seeing overall phylogenetic patterns and habitat use. Fig. 2 (particularly the new revised version) counts the number of loss events per gene across the phylogeny and uses resampling statistics to determine whether clock genes are lost more or less frequently than would be expected by chance. Fig. 3 shows evidence of specific gene loss events, including the observed mutations and patterns of synteny for lost genes, which we believe is an important component of the data. Finally, in the revised Fig. 4, we apply evolutionary statistics to track patterns of gene and habitat evolution across the tree, providing statistical support for convergent evolution in gene loss and habitat use.

The maximum percentage of intact coding sequence across all transcript isoforms does not strike me as a relevant metric as early frameshifts can render a transcript non-functional and the amount of functional sequence does not translate to functional protein domains. I recommend removing this portion of the main and supplementary figures and only showing the mutational variants across the genes. There are also numerous tools to quantify the impacts of variants that could be used to

quantify the impacts of frameshifts that don't result in premature stop codons but may remove functionally relevant regions.

> We disagree that the percentage of intact CDS is not a relevant metric. The intactness metric plays a key role in the TOGA classification system, and so reporting this data allows us to display the most intact version of each gene across all isoforms in one plot, where showing specific mutation patterns for the 100 species in our dataset is unwieldy. We do agree that some truncating variants may be more important than others (by removing key domains etc). However, for comparative genomics, relying on individual truncating variants is risky. More on this is discussed in response to the first 10%/last 10% comment below. More information is always a good idea though, so we have provided a new supplemental table (See reviewer 3 comment #2) that lists the observed truncating variants per gene.

It should be possible to jointly analyze depth and latitude...

> This is a good idea, and it is accurate that MCMCglmm allows for multiple predictor variables. The challenge in our analysis is that depth and latitude are both proxies for photoperiod (zeitgeber) and though they both impact hours of light, their impact is different, with latitude being highly seasonal oscillating between 24hrs light and dark, and depth being constant dark. Additionally, as we discuss in the manuscript, depth data from fisheries is notoriously noisy compared to latitudinal data, so mixing them would introduce more uncertainty to the latitudinal dataset. For these reasons, we believe the more biologically relevant and statistically viable approach is to model depth and latitude separately.

Figure 3D and 3F has several genes that are colored different colors (e.g. below dbx1 in pink), those genes are not defined anywhere and it's not clear why they are below dbx1 if they are not that gene. There should be another column for other genes with their gene names. If space is an issue, the assembly scaffold names could be provided in a supplementary table instead of within the figure.

> We agree this plotting was confusing. The original synteny approach plotted the nearest neighboring genes but not necessarily whether they were the same set of genes. Occasionally new genes (the cryptically labeled ones mentioned here) appear in the locus. We have modified this to pick up a set of upstream and downstream genes in the first species being plotted and to plot only these genes in subsequent species. The resulting synteny plots are below:
>
> **Revised Fig. 3:**

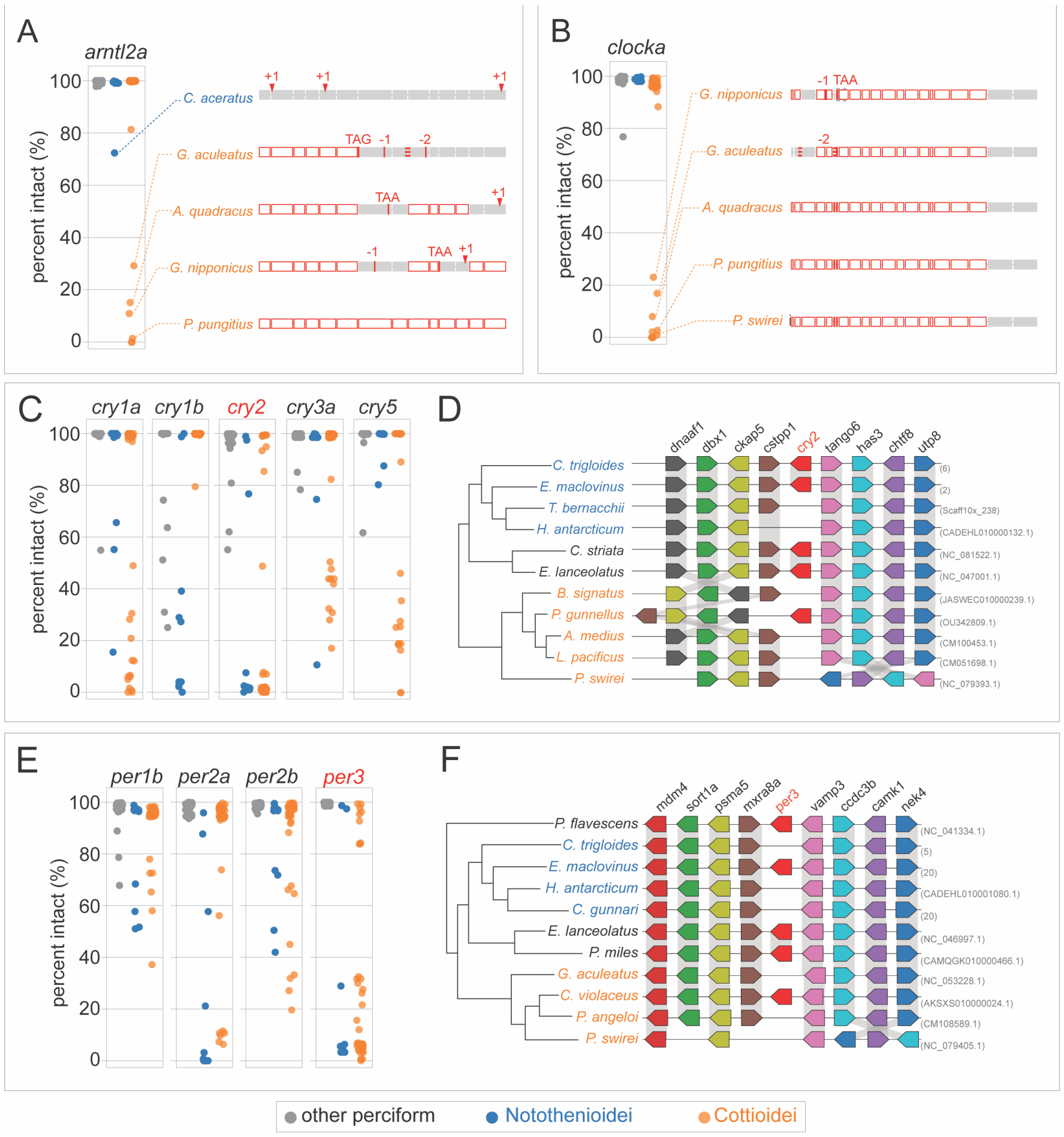

I would argue that the manuscript does not provide evidence that "reduced dependence on circadian systems may have contributed to their success as one of the most species-rich vertebrate orders". I recognize that this is a very general statement and includes "may". I agree that there is evidence that the losses "occurred early in their [the lineages] diversification".

> We have edited the abstract to rephrase this as a question rather than conditional statement.

> "Biological clocks allow organisms to anticipate cyclical environmental changes, yet in high-latitude or deep-sea habitats, the diel cues that entrain these rhythms are often seasonally diminished or absent. Fishes of the order Perciformes have rapidly diversified across these arrhythmic ecosystems, raising the question of whether changes to circadian rhythms and biological clock genetic architecture are a component of their evolutionary success. Here, we used a comparative genomic approach to investigate patterns of core biological clock gene loss across 96 perciform and five outgroup species. We found widespread and lineage-specific

loss in core clock genes, particularly in the convergently evolving polar and deep-sea suborders Notothenioidei and Cottioidei. This trend of circadian gene loss was significantly associated with higher-latitude habitat use along a latitudinal gradient. To determine if these genomic signatures reflect a functional loss of rhythmicity, we performed metabolic phenotyping on three notothenioid species. We found a consistent lack of circadian metabolic oscillations during the late austral fall across all notothenioids, including the sub-Antarctic sister lineage to the cryonotothenioid adaptive radiation, *Eleginops maclovinus*. Experimental data across Perciformes, combined with suborder-wide patterns of gene loss, suggest that a release from circadian constraints occurred early in their diversification, potentially facilitating the repeated expansion of these fishes into polar and deep-sea habitats."

It is possible to measure relaxed selection, for example using RELAX or other dn/ds methods which would add substantial analyses to this manuscript to quantify whether other (non-lost) circadian genes show relaxation of selection in deep sea lineages.

This is a great idea. Please see response to comment 3 from reviewer 3 for details. Briefly, inconsistent results depending on isoform usage combined with few new genes being significant which were not already exhibiting patterns of loss led us to decide not to include this statistic.

There does not seem to be directionality in TOGA for where mutations are outside of the middle 80% of a coding sequence, however mutations in the first 10% are likely to have a much larger impact than those at the final 10%.

This is an excellent point, and there are many studies in model systems that specifically target an early exon when making knockouts with tools like CRISPR/Cas9. However, in a comparative genomics scenario, these early frameshifts may also reflect changes to gene annotation between species, rendering the early frameshift effectively neutral. The authors of TOGA compared orthologs between humans and mice and found that 62% of frame shifts and premature stop codons identified by TOGA occurred in the first or last 10% of genes, which was attributed to the use of alternative start and stop codon usage in different species (see figure below from Kirilenko et al. 2023). Thus, while there can be mutations that prevent gene expression in the first or last 10% of the CDS, there are also a relatively high percentage of likely false positives. Conservatively, TOGA only classifies a gene as lost if multiple gene-inactivating mutations occur in the middle 80%, a condition that is very rarely found in genes that produce a functional protein. We also only present a gene with a status of "lost" if TOGA found it to be lost when compared to both of our reference genomes, which further mitigates reference bias in gene annotation.

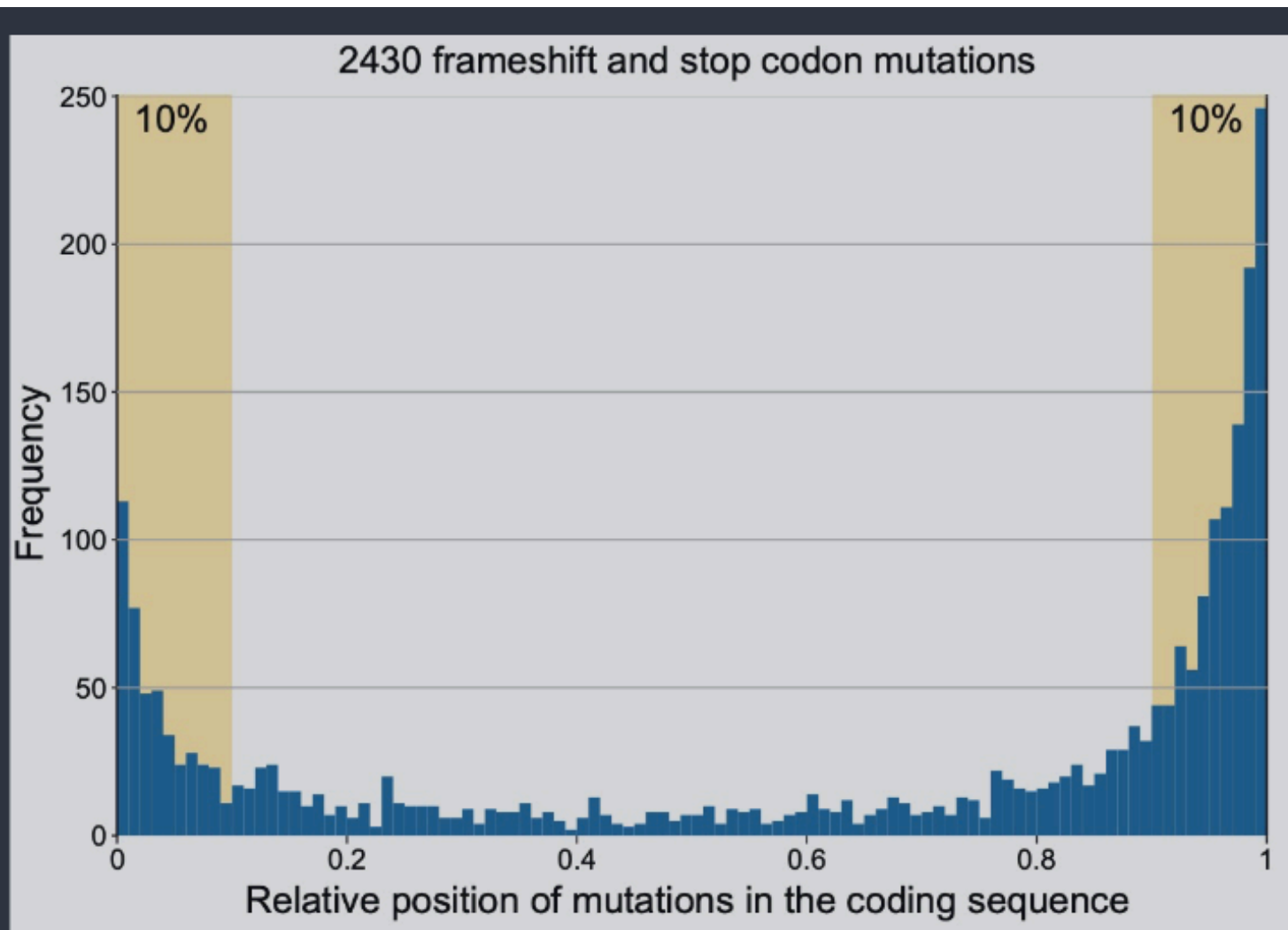

Fig. S12: Relative position of a total of 2,430 frameshift and stop codon mutations that TOGA detected in the coding sequence of human-mouse conserved genes.
Only 1:1 human-mouse orthologous genes according to the Ensembl were analyzed. The histogram shows that the relative position of 2,016 frameshifts and 414 premature stop codons (detected in 1,684 exons) is heavily biased toward the N-terminal and in particular to the C-terminal end of the coding region. In total, 62% of all frameshifts and stop codons occurred in the first or last 10% of CDS.

The light/dark cycles for the metabolic study are not reported. Was the light/dark the fall conditions or was there any artificial light (or none) provided during the study?

This is a good point. We have added the light/dark cycles for the study to methods:

> "We measured the circadian rhythm of the metabolic rate using an automatic intermittent-flow aquatic respirometry system. The respirometry system measured four individuals simultaneously, each within their own chamber. Hence, the metabolic profiles were obtained for every individual. The automation features of the system enabled undisturbed and continuous measurements for approximately 48 hours. To avoid the effects of digestion, spontaneous activities, and movement, the animals were fasted for 48 hours to reach a post-absorptive state. The metabolic rate of the animals was then measured in a dark and quiescent environment. Thus, only the innate rhythm was manifest in the metabolic profiles. The details of the metabolic rate calculation and the metabolic measuring techniques can be found below and in previously published studies using the same type of respirometry system (99–101). Before the experiment, animals were held in a ~12L:12D cycle in a tank that had covers to reduce visual disturbances, but the complete blocking of light was not guaranteed."
