# Supplementary material for: Convergent latitudinal erosion of circadian systems in a rapidly diversifying order of fishes
Source: PLoS Genet. 2026 Aug 31;22(8):e1012287. doi: 10.1371/journal.pgen.1012287 (PMC13557501; doi:10.1371/journal.pgen.1012287)
Supplement: Peer review history

# Peer review: Decision Letter 0

**DOI:** 10.1371/journal.pgen.1012287.r001

**Authors:** Josep M Comeron, Monica P. Colaiácovo

16 Apr 2026

PGENETICS-D-26-00009

Convergent latitudinal erosion of circadian systems in a rapidly diversifying order of fishes

PLOS Genetics

Dear Dr. Daane,

Thank you for submitting your manuscript to PLOS Genetics. After careful consideration, we feel that it has merit but does not fully meet PLOS Genetics's publication criteria as it currently stands. Therefore, we invite you to submit a revised version of the manuscript that addresses the points raised during the review process.

As you will see, the reviewers, all experts in the field, agreed that this is a strong study that will be of general interest. They also value the combination of large-scale phylogenomic analyses and detailed physiological studies. However, they also have a series of concerns and requests that should be addressed before acceptance.

Please submit your revised manuscript by July 15. If you will need more time than this to complete your revisions, please reply to this message or contact the journal office at plosgenetics@plos.org. When you're ready to submit your revision, log on to https://www.editorialmanager.com/pgenetics/ and select the 'Submissions Needing Revision' folder to locate your manuscript file.

Please include the following items when submitting your revised manuscript:

* A letter that responds to each point raised by the editor and reviewer(s). You should upload this letter as a separate file labeled 'Response to Reviewers'. This file does not need to include responses to any formatting updates and technical items listed in the 'Journal Requirements' section below.

* A marked-up copy of your manuscript that highlights changes made to the original version. You should upload this as a separate file labeled 'Revised Manuscript with Track Changes'.

* An unmarked version of your revised paper without tracked changes. You should upload this as a separate file labeled 'Manuscript'.

If you would like to make changes to your financial disclosure, competing interests statement, or data availability statement, please make these updates within the submission form at the time of resubmission. Guidelines for resubmitting your figure files are available below the reviewer comments at the end of this letter.

We look forward to receiving your revised manuscript.

Kind regards,

Josep M Comeron

Academic Editor

PLOS Genetics

Monica Colaiácovo

Section Editor

PLOS Genetics

Aimée Dudley

Editor-in-Chief

PLOS Genetics

Anne Goriely

Editor-in-Chief

PLOS Genetics

**Journal Requirements:**

1) Please ensure that the CRediT author contributions listed for every co-author are completed accurately and in full.

At this stage, the following Authors/Authors require contributions: Daniel Wright, Yangfan Zhang, and Jacob Daane. Please ensure that the full contributions of each author are acknowledged in the "Add/Edit/Remove Authors" section of our submission form.

The list of CRediT author contributions may be found here: https://journals.plos.org/plosgenetics/s/authorship#loc-author-contributions

2) We ask that a manuscript source file is provided at Revision. Please upload your manuscript file as a .doc, .docx, .rtf or .tex. If you are providing a .tex file, please upload it under the item type u2018LaTeX Source Fileu2019 and leave your .pdf version as the item type u2018Manuscriptu2019.

3) Please provide an Author Summary. This should appear in your manuscript between the Abstract (if applicable) and the Introduction, and should be 150-200 words long. The aim should be to make your findings accessible to a wide audience that includes both scientists and non-scientists. Sample summaries can be found on our website under Submission Guidelines:

https://journals.plos.org/plosgenetics/s/submission-guidelines#loc-parts-of-a-submission

4) We do not publish any copyright or trademark symbols that usually accompany proprietary names, eg ©,  ®, or TM  (e.g. next to drug or reagent names). Therefore please remove all instances of trademark/copyright symbols throughout the text, including:

- ® on page: 11.

5) Please upload all main figures as separate Figure files in .tif or .eps format. For more information about how to convert and format your figure files please see our guidelines:

https://journals.plos.org/plosgenetics/s/figures

6) We have noticed that you have uploaded Supporting Information files, but you have not included a list of legends. Please add a full list of legends for your Supporting Information files after the references list.

7) Please amend your detailed Financial Disclosure statement. This is published with the article. It must therefore be completed in full sentences and contain the exact wording you wish to be published.

- State the initials, alongside each funding source, of each author to receive each grant. For example: "This work was supported by the National Institutes of Health (####### to AM; ###### to CJ) and the National Science Foundation (###### to AM)."

- State what role the funders took in the study. If the funders had no role in your study, please state: "The funders had no role in study design, data collection and analysis, decision to publish, or preparation of the manuscript.".

If you did not receive any funding for this study, please simply state: u201cThe authors received no specific funding for this work.u201d

**Reviewers' comments:**

Reviewer's Responses to Questions

**Comments to the Authors:**

Reviewer #1:

In this manuscript, Wright et al explore an interesting hypothesis, that Perciformes fish species which have adapted to deep sea or high lattitude environments with no or little circadian rhythm would have lost genes related to circadian clock regulation. For this, they characterise orthologs of core circadian clock genes in perciform genomes. They indeed find loss of circadian genes with high lattitude, but surprisingly not with deep sea environment.

I appreciated the interesting question, and the detailed analysis of gene loss rather than a simple "no Blast hit", which provides insight into the process of pseudogeneisation. The authors also nicely confirm the loss of physiological circadian rhythm in high lattitude notothenioids, in relation to gene loss.

Major comments:

1- Orthology results are central to the work, yet only one orthology method is used. Although it is a good method, and well adapted, I suggest confirming the robustness of results with at least one protein similarity graph method, and reporting orthology results from public databases such as Ensembl Compara, EggNOGG, OrthoDB or OMA. In addition to limiting bias from the choice of method, this would limit bias due to the choice of two specific reference genomes.

2- I have several comments on the comparison between species and phylogenetic reasoning. Gene loss in the ancestor of a species clade is one single event, which mostly cannot be reversed. Thus:

2a- Gene losses should not be counted in number of genomes without a gene, but in number of monophyletic clades without a gene, which correspond to the number of events of loss. This notably applies to Fig. 2, and to the paragraph starting line 155.

2b- For Fig. 4, what biology are the authors representing with a model "which allows for backward and forward transitions"? A more straighforward interpretation of the tree is that each clade with only losses corresponds to one loss event, and every clade with a gene present in even one species implies that the gene was present in the ancestor of this clade. It would be more informative to map on the tree of Fig 4 the number of circadian genes infered lost on each branch.

2c- The randomisation around line 198 doesn't take into account phylogenetic non-independence. Instead of randomising genomes, the authors should randomise branches on the tree on which losses can occur.

2d- The authors could use CAFE5 (https://doi.org/10.1093/bioinformatics/btaa1022) to evaluate significant patterns of gain and loss.

3- Potential losses of function can sometimes be solved with gene expression. For example stickleback Per1b has expression in the database Bgee (https://doi.org/10.1093/nar/gkae1118). The authors should consider checking these potential losses in expression datasets or databases.

4- The first paragraph of the Discussion is ambiguous about drift or adaptation ("likely a key component of their success"). Unless there is evidence of adaptation, the default assumption should be that these genes were lost by drift, in the absence of purifying selection maintaining their function in these environments.

5- While the comparison to cave species is interesting, the authors should note that these species have evolved for much shorter times in their extreme environments than the high lattitude clades have, and with much smaller population sizes.

6- Fig. 2 shows indentical one-sided and two-sided p-values, which is impossible. Moreover, there is no strong reason to use one-sided tests, i.e. an excess in the other direction would also have been of interest. The authors should use only two-sided tests. See e.g. https://doi.org/10.1111/j.1442-9993.2009.01946.x, https://doi.org/10.1111/j.2041-210X.2010.00014.x.

7- Please provide the MCMCglmm model diagnostic plots and autocorrelation plots.

8a- In Table S2, please provide BUSCO or equivalent scores for the genomes, which is important for this type of study of gene loss.

8b- Statistics are missing for H. otakii.

Minor comments:

9- It is worth noting that Ref 42 Toloza-Villalobos et al which is discussed several times did not consider all of the genes which are studied here, e.g. they don't consider Bmal.

10- It would be informative to show transitions to high lattitude or deep sea on the branches of trees of figs. 1 and 4.

Reviewer #2: This manuscript provides a large-scale analysis of evolved changes in circadian clock genes across Perciforms. The broader question about whether clock genes are lost is particularly interesting. Circadian clocks are widely believe to be nearly ubiquitous. This manuscript examines a relatively comprehensive list of circadian genes across 96 Perciform species. The paper is of significance and of broad interest, given that it provides the most throrough analysis to date of clock-gene loss. The paper also presents ambitious metabolic profiling of ice fish, showing a functional loss of rhythms. The manuscript is well-written and statistically sound. Below are a number of suggestions that would help increase the accessibility to a wider audience, particularly the circadian community.

1. The paper would benefit from greater discussion the role of clock genes outside of Zeitgeber regulation (non-circadian). Many have functions that differ from their role in in daily rhythms. Presumably loss of clock-genes has wide-ranging effects on physiological and cellular function.

2. Why would a species lose seven circadian genes? Are there shared or partial functions? Presumably loss of one, or a few, key genes can fully disable the clock. If there is a way to extend the analysis in 2E,F to discuss the broader functions of the circadian clock? Could the analysis be extended to compare core-clock genes vs output-associated genes?

3. Some reorganization and clarification of jargon would make this more accessible to a generalist audience. For example, see paragraph at line 225.

4. Could the authors discuss on changes in circadian genes in highly divergent subterranean animals with loss of circadian genes including mole rates and cavefish?

5. It would be helpful to discuss why loss of clock genes might be advantageous, and particularly how the loss of specific Zeitgebers may push adaptive pressure towards loss of the clock.

Reviewer #3: **Please see attachment.**

Reviewer #4: This manuscript explores the loss of circadian related genes in numerous perciform fishes . This manuscript combines comparative genomics of circadian genes and metabolic phenotyping of 4 species (2 polar, 1 nearly polar, and 1 non-polar). This manuscript does a nice job of combining a detailed comparative genomics study with data on several species. I enjoyed reading this manuscript. My apologies for the delay in returning the review.

The figures 1-4 seem to have somewhat redundant information. The information is displayed nicely but it’s unclear to me how different the information is that is being displayed in each figure.

The maximum percentage of intact coding sequence across all transcript isoforms does not strike me as a relevant metric as early frameshifts can render a transcript non-functional and the amount of functional sequence does not translate to functional protein domains. I recommend removing this portion of the main and supplementary figures and only showing the mutational variants across the genes. There are also numerous tools to quantify the impacts of variants that could be used to quantify the impacts of frameshifts that don’t result in premature stop codons but may remove functionally relevant regions.

It should be possible to jointly analyze depth and latitude…

Figure 3D and 3F has several genes that are colored different colors (e.g. below dbx1 in pink), those genes are not defined anywhere and it’s not clear why they are below dbx1 if they are not that gene. There should be another column for other genes with their gene names. If space is an issue, the assembly scaffold names could be provided in a supplementary table instead of within the figure.

I would argue that the manuscript does not provide evidence that “reduced dependence on circadian systems may have contributed to their success as one of the most species-rich vertebrate orders”. I recognize that this is a very general statement and includes "may". I agree that there is evidence that the losses “occurred early in their [the lineages] diversification”.

It is possible to measure relaxed selection, for example using RELAX or other dn/ds methods which would add substantial analyses to this manuscript to quantify whether other (non-lost) circadian genes show relaxation of selection in deep sea lineages.

There does not seem to be directionality in TOGA for where mutations are outside of the middle 80% of a coding sequence, however mutations in the first 10% are likely to have a much larger impact than those at the final 10%.

The light/dark cycles for the metabolic study are not reported. Was the light/dark the fall conditions or was there any artificial light (or none) provided during the study?

**********

**Have all data underlying the figures and results presented in the manuscript been provided?**

Large-scale datasets should be made available via a public repository as described in the *PLOS Genetics*  data availability policy, and numerical data that underlies graphs or summary statistics should be provided in spreadsheet form as supporting information.

Reviewer #1: Yes

Reviewer #2: Yes

Reviewer #3: Yes

Reviewer #4: **No:**  The data for the metabolic study do not appear to be provided in the supporting information.

**********

PLOS authors have the option to publish the peer review history of their article (what does this mean?). If published, this will include your full peer review and any attached files.

If you choose “no”, your identity will remain anonymous but your review may still be made public.

**Do you want your identity to be public for this peer review?**  For information about this choice, including consent withdrawal, please see our Privacy Policy.

Reviewer #1: No

Reviewer #2: No

Reviewer #3: No

Reviewer #4: No

[NOTE: If reviewer comments were submitted as an attachment file, they will be attached to this email and accessible via the submission site. Please log into your account, locate the manuscript record, and check for the action link "View Attachments". If this link does not appear, there are no attachment files.]

**Figure resubmission:**

**Reproducibility:**

To enhance the reproducibility of your results, we recommend that authors of applicable studies deposit laboratory protocols in protocols.io, where a protocol can be assigned its own identifier (DOI) such that it can be cited independently in the future. Additionally, PLOS ONE offers an option to publish peer-reviewed clinical study protocols. Read more information on sharing protocols at https://plos.org/protocols?utm_medium=editorial-email&utm_source=authorletters&utm_campaign=protocols

Attachment Submitted filename: Wright et al.pdf

# Author comment: Author response to Decision Letter 1

**DOI:** 10.1371/journal.pgen.1012287.r002

17 Jun 2026

Attachment Submitted filename: Reviewer_comments-3.pdf

# Peer review: Decision Letter 1

**DOI:** 10.1371/journal.pgen.1012287.r003

**Authors:** Josep M Comeron, Monica P. Colaiácovo

17 Aug 2026

Dear Dr Daane,

Thank you for submitting a revised version of the study that has addressed the reviewers' comments and concerns. We are pleased to inform you that your manuscript entitled "Convergent latitudinal erosion of circadian systems in a rapidly diversifying order of fishes" has been editorially accepted for publication in PLOS Genetics. Congratulations!

Before your submission can be formally accepted and sent to production you will need to complete our formatting changes, which you will receive in a follow up email. Please be aware that it may take several days for you to receive this email; during this time no action is required by you. Please note: the accept date on your published article will reflect the date of this provisional acceptance, but your manuscript will not be scheduled for publication until the required changes have been made.

Once your paper is formally accepted, an uncorrected proof of your manuscript will be published online ahead of the final version, unless you’ve already opted out via the online submission form. If, for any reason, you do not want an earlier version of your manuscript published online or are unsure if you have already indicated as such, please let the journal staff know immediately at plosgenetics@plos.org.

In the meantime, please log into Editorial Manager at https://www.editorialmanager.com/pgenetics/, click the "Update My Information" link at the top of the page, and update your user information to ensure an efficient production and billing process. Note that PLOS requires an ORCID iD for all corresponding authors. Therefore, please ensure that you have an ORCID iD and that it is validated in Editorial Manager. To do this, go to ‘Update my Information’ (in the upper left-hand corner of the main menu), and click on the Fetch/Validate link next to the ORCID field.  This will take you to the ORCID site and allow you to create a new iD or authenticate a pre-existing iD in Editorial Manager.

If you have a press-related query, or would like to know about making your underlying data available (as you will be aware, this is required for publication), please see the end of this email. If your institution or institutions have a press office, please notify them about your upcoming article at this point, to enable them to help maximise its impact. Inform journal staff as soon as possible if you are preparing a press release for your article and need a publication date.

Thank you again for supporting open-access publishing; we are looking forward to publishing your work in PLOS Genetics!

Yours sincerely,

Josep M Comeron

Academic Editor

PLOS Genetics

Monica Colaiácovo

Section Editor

PLOS Genetics

Aimée Dudley

Editor-in-Chief

PLOS Genetics

Anne Goriely

Editor-in-Chief

PLOS Genetics

www.plosgenetics.org

BlueSky: @plos.bsky.social

----------------------------------------------------

Comments from the reviewers (if applicable):

Reviewer's Responses to Questions

**Comments to the Authors:**

**Please note here if the review is uploaded as an attachment.**

Reviewer #1: I thank the authors for the excellent job they did in this revision. The revised manuscript is a pleasure to read.

One small comment: p. 5, it should probably be "expected mean" in the parentheses rather than just "mean".

**********

**Have all data underlying the figures and results presented in the manuscript been provided?**

Large-scale datasets should be made available via a public repository as described in the *PLOS Genetics*  data availability policy, and numerical data that underlies graphs or summary statistics should be provided in spreadsheet form as supporting information.

Reviewer #1: Yes

**********

PLOS authors have the option to publish the peer review history of their article (what does this mean?). If published, this will include your full peer review and any attached files.

If you choose “no”, your identity will remain anonymous but your review may still be made public.

**Do you want your identity to be public for this peer review?**  For information about this choice, including consent withdrawal, please see our Privacy Policy.

Reviewer #1: No

----------------------------------------------------

**Data Deposition**

If you have submitted a Research Article or Front Matter that has associated data that are not suitable for deposition in a subject-specific public repository (such as GenBank or ArrayExpress), one way to make that data available is to deposit it in the Dryad Digital Repository. As you may recall, we ask all authors to agree to make data available; this is one way to achieve that. A full list of recommended repositories can be found on our website.

The following link will take you to the Dryad record for your article, so you won't have to re‐enter its bibliographic information, and can upload your files directly:

http://datadryad.org/submit?journalID=pgenetics&manu=PGENETICS-D-26-00009R1

More information about depositing data in Dryad is available at http://www.datadryad.org/depositing. If you experience any difficulties in submitting your data, please contact help@datadryad.org for support.

Additionally, please be aware that our data availability policy requires that all numerical data underlying display items are included with the submission, and you will need to provide this before we can formally accept your manuscript, if not already present.

----------------------------------------------------

**Press Queries**

If you or your institution will be preparing press materials for this manuscript, or if you need to know your paper's publication date for media purposes, please inform the journal staff as soon as possible so that your submission can be scheduled accordingly. Your manuscript will remain under a strict press embargo until the publication date and time. This means an early version of your manuscript will not be published ahead of your final version. PLOS Genetics may also choose to issue a press release for your article. If there's anything the journal should know or you'd like more information, please get in touch via plosgenetics@plos.org.

# Editor report: Acceptance letter

**DOI:** 10.1371/journal.pgen.1012287.r004

**Authors:** Josep M Comeron, Monica P. Colaiácovo

PGENETICS-D-26-00009R1

Convergent latitudinal erosion of circadian systems in a rapidly diversifying order of fishes

Dear Dr Daane,

We are pleased to inform you that your manuscript entitled "Convergent latitudinal erosion of circadian systems in a rapidly diversifying order of fishes" has been formally accepted for publication in PLOS Genetics! Your manuscript is now with our production department and you will be notified of the publication date in due course.

The corresponding author will soon be receiving a typeset proof for review, to ensure errors have not been introduced during production. Please review the PDF proof of your manuscript carefully, as this is the last chance to correct any errors. Please note that major changes, or those which affect the scientific understanding of the work, will likely cause delays to the publication date of your manuscript.

Soon after your final files are uploaded, unless you have opted out or your manuscript is a front-matter piece, the early version of your manuscript will be published online. The date of the early version will be your article's publication date. The final article will be published to the same URL, and all versions of the paper will be accessible to readers.

For Research Articles, you will receive an invoice from PLOS for your publication fee after your manuscript has reached the completed accept phase. If you receive an email requesting payment before acceptance or for any other service, this may be a phishing scheme. Learn how to identify phishing emails and protect your accounts at https://explore.plos.org/phishing.

Thank you again for supporting PLOS Genetics and open-access publishing. We are looking forward to publishing your work!

With kind regards,

Anitha Samidurai

PLOS Genetics

On behalf of:

The PLOS Genetics Team

Carlyle House, Carlyle Road, Cambridge CB4 3DN | United Kingdom

plosgenetics@plos.org | +44 (0) 1223-442823

plosgenetics.org | Twitter: @PLOSGenetics
